# Pleiotropic role of PAX cyclolipopeptides in the *Xenorhabdus* bacterium mutualistically associated with entomopathogenic nematodes

**DOI:** 10.1128/aem.00760-25

**Published:** 2025-09-09

**Authors:** Noémie Claveyroles, Anne Lanois-Nouri, Imane El Fannassi, Jean-Claude Ogier, Sylvie Pagès, Adrien Chouchou, Guillaume Cazals, Gilles Valette, Alyssa Carré-Mlouka, Alain Givaudan

**Affiliations:** 1 DGIMI, Université de Montpellier, INRAE 27057, Montpellier, France; 2 Institut des Biomolécules Max Mousseron (IBMM), Pôle Chimie Balard Recherche, Université de Montpellier, CNRS, ENSCM, Montpellier, France; 3 Plateforme d’Analyses et de Caractérisations Chimie Balard, Université de Montpellier, CNRS, ENSCM, Montpellier, France; UMR Processus Infectieux en Milieu Insulaire Tropical, Ste Clotilde, France

**Keywords:** *Xenorhabdus*, *Steinernema*, NRPS, specialized metabolites, biofilm, swimming motility, mutualism

## Abstract

*Xenorhabdus* is an entomopathogenic bacterium involved in a mutualistic relationship with *Steinernema* nematodes. *Xenorhabdus* produces a multitude of specialized metabolites by non-ribosomal peptide synthetase (NRPS) pathways to mediate bacterium-nematode-insect interactions. PAX cyclolipopeptides are a family of NRP-type molecules whose ecological role remains poorly understood. In this study, the pleiotropic role of PAX peptides in the life cycle of *Xenorhabdus nematophila* has been investigated. By mass spectrometry analysis, we first demonstrated that PAX peptides were detected from the pathogenic stage up to the necrotrophic stage. We discovered that the bromothymol blue adsorption phenotype historically used to discriminate *Xenorhabdus* variants was associated with the presence of PAX peptides. We found that PAX peptides were positively involved in biofilm formation and negatively involved in swimming motility. PAX peptides were also shown to promote *in vivo* the production of infective *Steinernema* juveniles, suggesting their involvement in the mutualistic relationship between *Xenorhabdus* and its nematode partner. Finally, we showed that the *paxTABC* cluster, as well as PAX peptide production, was conserved across the whole *Xenorhabdus* genus except in *Xenorhabdus poinarii* and *Xenorhabdus ishibashii*. This work has revealed multiple new ecological roles for NRP-type peptides.

**IMPORTANCE:**

*Xenorhabdus* bacteria are models of particular interest for their mutualistic relationship with *Steinernema* nematodes and their ability to produce a wide range of natural NRP-type bioactive metabolites. These compounds are mostly studied for their medical or industrial applications, but their ecological role is poorly understood. This study provides a dynamic characterization of PAX cyclolipopeptide presence during the *Xenorhabdus nematophila* life cycle, as well as confirmation of their production by seven different strains within the *Xenorhabdus* genus. We revealed new multiple functions for PAX peptides in biofilm formation, swimming motility, and juvenile nematode production. A deeper understanding of how PAX peptides interact with the nematode host would provide a better insight into the role of these cyclolipopeptides in bacterial-nematode mutualism.

## INTRODUCTION

Entomopathogenic bacteria of the genus *Xenorhabdus* (*Morganellaceae*) are hosted by nematodes of the *Steinernema* genus, with which they have co-evolved to establish long-lasting, mutually beneficial interactions (1). *Xenorhabdus* are carried into the insect larvae by the infective juvenile (IJ) stage of nematodes. IJs live in the soil, seeking insect larvae to infest. Once they have found a prey, IJs penetrate insect larvae through their natural orifices and release their *Xenorhabdus* bacteria into the insect hemocoel (2). Bacteria cause septicemia and death of the insect larvae within 24–48 h, enabling the nematodes to use the insect cadaver as a nutritive resource and a host for their development (3). Several nematode reproduction cycles occur successively inside the cadaver (4). During this necrotrophic stage, *Xenorhabdus* and *Steinernema* nematodes interact with a range of microorganisms from the nematode microbiota (frequently associated microbiota, FAM) and the insect microbiota (5–7). When nutrients are depleted, mutualistic bacteria and nematodes reassociate, and IJs emerge in the soil seeking a new host. Entomopathogenic nematodes are considered promising biological control agents of key insect pests (8).

To cope with changing conditions during its complex life cycle, *Xenorhabdus* can produce a wide variety of specialized metabolites, including non-ribosomal peptides (NRPs) (9, 10). Non-ribosomal peptide synthetases (NRPSs) are modular enzymes that catalyze the synthesis of a wide range of peptides from a variety of proteinogenic and non-proteinogenic amino acid substrates. In *Xenorhabdus nematophila*, the *ngrA* gene encodes the phosphopantetheinyl transferase (PPTase), an enzyme that activates NRPS by transferring a cofactor (4′-phosphopantetheine), thus enabling the production of NRP-type metabolites (11). It has been shown that *ngrA*-dependent compounds are responsible for most of the antimicrobial activity of *X. nematophila* and are required to eliminate bacterial competitors *in vitro* (12). It has also been demonstrated that *ngrA*-dependent compounds from *X. nematophila* are required for optimal growth and development of their nematode partner *in vivo*. Indeed, *Steinernema carpocapsae* reproduction was reduced in insects infected with an *ngrA* mutant (12). Similar results have been observed with *Photorhabdus luminescens* associated with the nematode *Heterorhabditis bacteriophora* (13).

Studies on NRP metabolites in *Xenorhabdus* have so far focused mainly on their antimicrobial potential (14). These include xenocoumacins, odilorhabdins, xenoamicins, xenematides, xenortides, rhabdopeptides, RXPs, GameXpeptides, nematophin, xenobactin, szentiamide, bicornitun, taxlllaids A-G, and PAX peptides (15–28). Some of these compounds also exhibit insecticidal (xenocoumacins [29], xenematides [18], RXPs, and [21]), antiprotozoal (xenoamicins [17], xenortides [19], RXPs [21], xenobactin [24], and taxlllaids A-G [27]), acaricide (xenocoumacins [30]), and anti-inflammatory (xenocoumacins [31]) properties.

Our study focuses on the cyclolipopeptides PAX (peptide antimicrobial from *Xenorhabdus*) family (28). NRPS enzymes involved in PAX peptide biosynthesis are encoded by the *paxTABC* gene cluster. The *paxA*, *paxB,* and *paxC* genes encode the three NRPS enzymes responsible for heptapeptide synthesis and cyclization. The *paxT* gene encodes a putative membrane transport protein suspected of exporting PAX peptides outside the cell. PAX cyclolipopeptides are cationic peptides containing seven amino acids, among which five lysines constitute a cycle, with an N-terminal fatty acid moiety (32). Different PAX peptides, with variations in the length of the fatty acid chain or the nature of the amino acid in position 2 (lysine or arginine), have been identified among different strains and species of *Xenorhabdus* (28, 32, 33). Bacterial specialized metabolites are widely investigated in the literature for their medical or agricultural applications, but their ecological roles are still poorly understood (34). PAX peptides were first studied for their antimicrobial activities against *Micrococcus luteus* and *Fusarium oxysporum* (28). PAX peptides exhibit no insecticidal activity against *Spodoptera littoralis*, *Galleria mellonella*, *Locusta migratoria*, *Manduca sexta,* and insect hemocytes, nor nematicidal activity against *Caenorhabditis elegans* (28, 35). As external addition of synthetic labeled PAX peptides to *Xenorhabdus doucetiae* resulted in their localization at the cell surface, it was suggested that PAX peptides may provide protection against cationic antimicrobial peptides (AMPs) produced by the insect via positive charge repulsion mechanisms (36).

In this study, we revealed by mass spectrometry that PAX peptides were detected from the pathogenic stage to the late necrotrophic stage of the *X. nematophila* life cycle. We therefore aimed to elucidate the role(s) of PAX peptides using two deletion mutants of *X. nematophila* F1, ∆*paxA* and ∆*ngrA* mutants (37). By comparing the two mutants with the wild-type (WT) strain, we demonstrated different phenotypic characteristics on agar media, in swimming motility, and in biofilm formation *in vitro*. By minimum inhibitory concentration (MIC) assays, we showed that PAXs were weakly antimicrobial toward natural competitors of *Xenorhabdus*. We also assessed the pathogenicity of bacteria against insect larvae. Using aposymbiotic nematodes reassociated with the WT strain or the ∆*paxA* or ∆*ngrA* mutants, we investigated the involvement of PAX peptides in nematode reproductive success *in vivo*. Finally, we have highlighted the distribution of *paxTABC* genes and detected PAX peptide production throughout the *Xenorhabdus* genus. These findings reveal that PAX peptides are highly conserved compounds playing multiple roles in the *Xenorhabdus* life cycle.

## RESULTS

### PAX peptides are mostly detected during the necrotrophic stage of the *X. nematophila* life cycle

To understand the importance of PAX peptides in the life cycle of *X. nematophila* F1, PAX peptide presence was investigated at each stage of the bacterial life cycle. We first characterized all PAX peptides produced in stationary-phase extracts of the WT strain in a 48-h liquid culture using MALDI-TOF-MS. Twelve PAX peptides previously identified in the literature by Fuchs et al. (32) were detected in stationary-phase extracts of the WT strain: PAX1′ (*m/z* = 1,052.772 Da), PAX3′ (1,066.787), PAX2′/PAX7 (1,080.783), PAX4′ (1,094.797), PAX6 (1,078.766), PAX8 (1,102.772), PAX9 (1,106.797), PAX10 (1,108.812), PAX11 (1,120.808), PAX12 (1,122.818), and PAX13 (1,134.828) (Table S1) (32).

**Fig 1:**
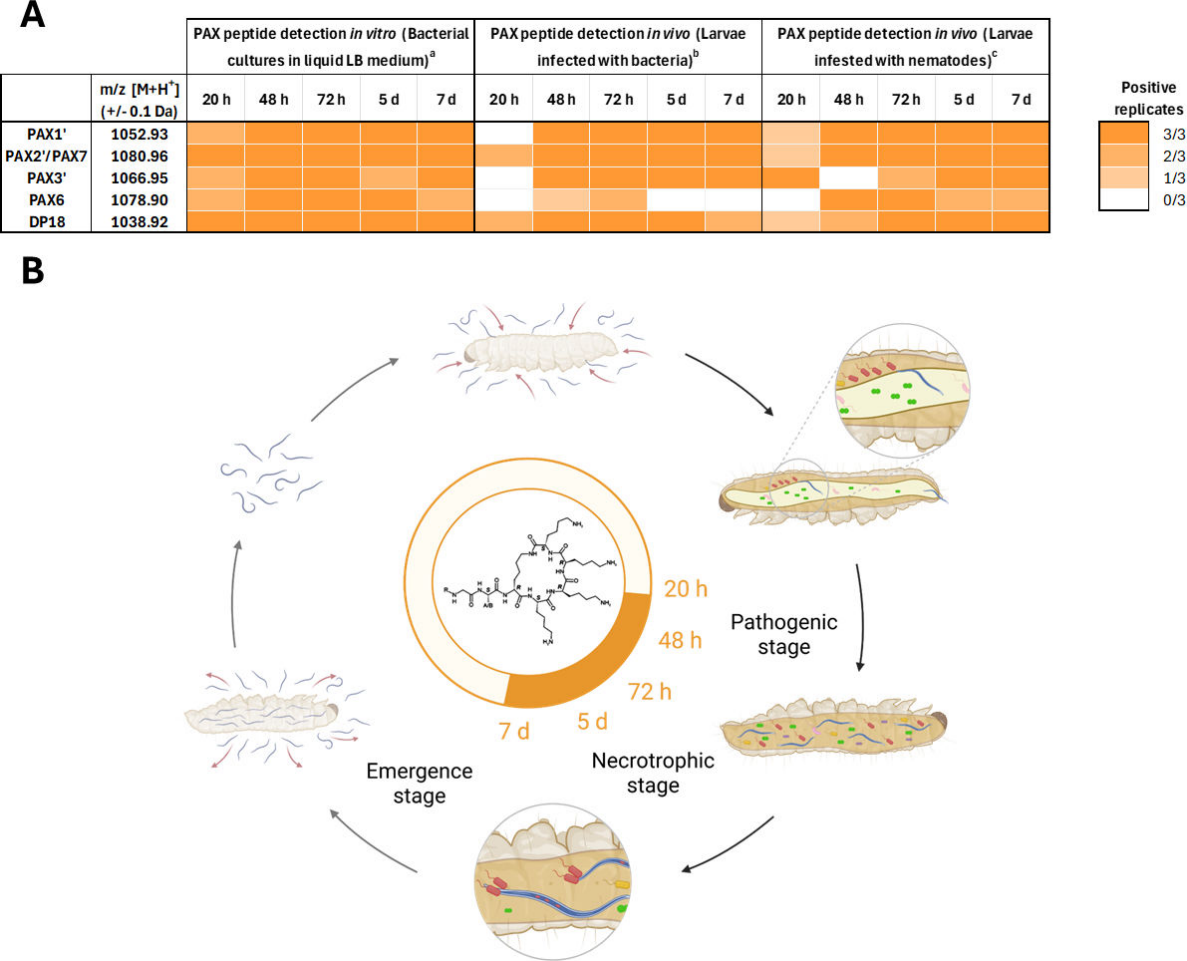
Presence of PAX peptides in extracts of *X. nematophila* WT grown *in vitro* and *in vivo*. (**A**) Detection by MALDI-TOF-MS analysis of selected PAX peptides (PAX1′, PAX2′/PAX7, PAX3′, and PAX6) and the extraction control DP18 in extracts of *X. nematophila* WT grown *in vitro* in liquid LB medium (a) and *in vivo* in 10 *G. mellonella* larvae injected with *X. nematophila* WT (b) or infested with *Steinernema carpocapsae* SK27 IJ nematodes (c). A synthetic PAX peptide derivative DP18 (*m/z* = 1,038.92 ± 0.1 Da) was added as a positive extraction control before the extraction steps. Experiments were performed in triplicate. Orange squares indicate the number of positive biological replicates in which the *m/z* value (±0.1 Da) of the corresponding PAX peptides was detected. The corresponding mass spectra are given in Table S2. (**B**) Visual representation of PAX peptide presence in different stages of the *Xenorhabdus* life cycle. The orange circle area represents the period of detection of PAX peptides. Figure was created with Biorender.

Then, the presence of most frequently detected PAX peptides (PAX1′, PAX2′/PAX7, PAX3′, and PAX6) was analyzed at 20 h, 48 h, 72 h, 5 days, and 7 days under three different experimental setups: (i) *in vitro* bacterial cultures, (ii) insect larvae infected with *X. nematophila* WT bacteria, (iii) insect larvae infested with *S. carpocapsae* SK27 nematodes carrying *X. nematophila* WT bacteria (Fig. 1; Table S2). An extraction control was performed by adding a synthetic PAX derivative (DP18, Fig. S1) before the extraction steps. The *m/z* values (±0.1 Da) of selected PAX peptides and the extraction control DP18 were screened by MALDI-TOF-MS analysis. In bacterial culture in lysogeny broth (LB) culture medium, PAX peptides were consistently found from 20 h (late exponential stage, Fig. S2) up to 7 days. Under *in vivo* conditions of *Galleria mellonella* larvae infected with bacteria or infested with nematodes, higher variability was observed at 20 h between biological samples. However, PAX peptides were detected from 48 h to 7 days in both *in vivo* conditions, except for PAX6, which is less frequently found in bacterial-infected larvae. These results demonstrate that PAX peptides are detected from the end of the pathogenic phase (20 h) to the late necrotrophic phase (7 days) of the *Xenorhabdus* life cycle.

### *paxA* mutation affects lecithinase-like activity, lipolytic activity, and bromothymol blue adsorption

**Fig 2:**
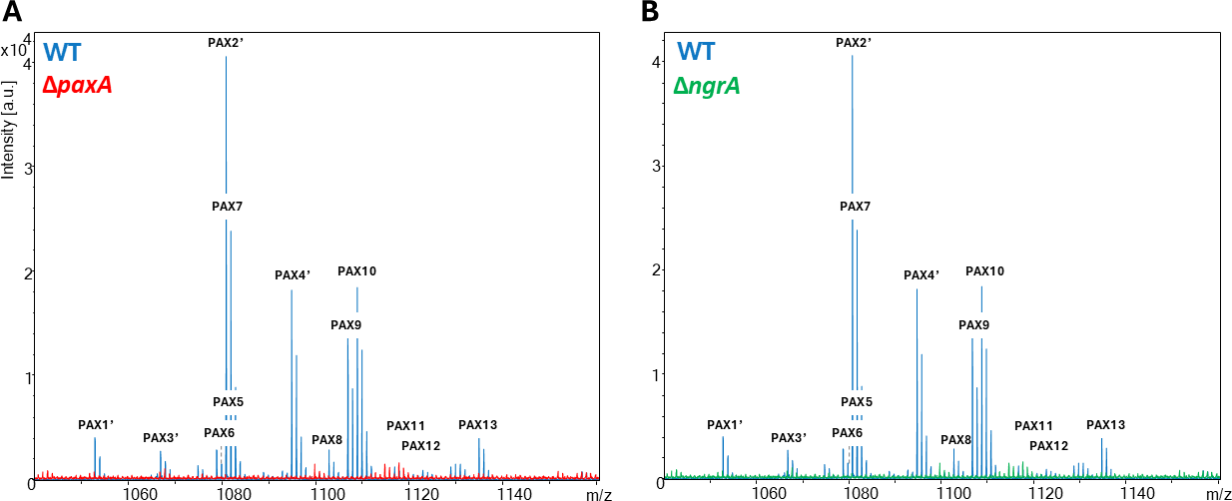
Presence of PAX peptides in extracts of *X. nematophila* WT (blue), ∆*paxA* (red), and ∆*ngrA* (green). (**A**) Merging of MALDI-TOF-MS mass spectra of extracts from 48 h stationary-phase cultures of WT and ∆*paxA* strains. (**B**) Merging of MALDI-TOF mass spectra of extracts from 48 h stationary-phase cultures of WT and ∆*ngrA* strains. The monoisotopic *m/z* values of PAX peptides are shown with their numbers previously identified by Fusch et al. (32). Peak heights represent intensity in arbitrary units (a.u). Exact *m/z* values (±0.05 Da) of each PAX peptide are listed in Table S1.

To assess the impact of PAX peptide production on phenotypic characteristics, a deletion mutant (∆*paxA*) was constructed in *X. nematophila* F1 by inserting an Ω-cam interposon by allelic exchange at the beginning of the *paxA* gene, the first NRPS gene in the *paxTABC* cluster. As phosphopantetheinyl transferase enzymes enable NRPS mega-synthetase activation, a PPTase-deficient mutant (∆*ngrA*) of *X. nematophila* F1 was also used as a global non-producer of NRP-type metabolites. The absence of PAX peptide production by ∆*paxA* (Fig. 2A) and ∆*ngrA* (Fig. 2B) mutants was confirmed by MALDI-TOF-MS in extracts from stationary-phase cultures.

The phenotypic traits of the ∆*paxA* and ∆*ngrA* mutants were then tested through various phenotypic assays on agar media. The ∆*paxA* and ∆*ngrA* mutants displayed enhanced lipolytic activity on Tween 20, 40, 60, 80, and 85 and suppressed lecithinase-like activity compared to the WT strain (Table 1). However, no difference in hemolysis was observed. As expected, the ∆*ngrA* mutant showed weaker antimicrobial activity against *M. luteus* than the WT strain or ∆*paxA* mutant. Surprisingly, both mutant strains displayed red colonies on Nutrient Bromothymol Blue Agar (NBTA) medium, indicating no bromothymol blue (BBT) adsorption (Fig. S3). These observations demonstrate that PAX peptides are responsible for BBT adsorption. Together, these results indicate that the mutation of Δ*paxA* and Δ*ngrA* induces pleiotropic effects on phenotypes of *X. nematophila* that are similar for both mutants, except for differences in antimicrobial activity against *M. luteus*.

**TABLE 1:** Phenotypic traits of *X. nematophila* WT, ∆*paxA,* and ∆*ngrA* on agar media

Phenotypic assay	WT	∆*paxA*	∆*ngrA*
Lipolysis^*a*^			
Tween 20, Tween 40, Tween 60, Tween 80	W+	+	+
Tween 85	W+	+	+
Lecithinase-like activity^*b*^	+	−	−
Antimicrobial activity (*Micrococcus luteus*)^*c*^	37.5	37.6	15.7
Hemolysis^*d*^	T	T	T
Adsorption of bromothymol blue and TTC degradation into formazan^*e*^	Blue	Red	Red

^*a*^ Precipitation of insoluble calcium salts around the colony associated with fatty acid hydrolysis (Tween 20, 40, 60, 80, or 85) after 72-h incubation. +, halo larger than 8 mm in diameter or brightening around the colony for Tween 85. W+, weak+, precipitate with a halo lower than 8 mm or weak brightening around the colony for Tween 85.

^*b*^ Precipitation of egg lecithin after 72-h incubation. +, opaque white halo around the colony associated with phospholipid precipitation. −, no halo, no phospholipid precipitation.

^*c*^ Antimicrobial activity against *M. luteus* after 48-h incubation. Halo diameter in millimeters.

^*d*^ Hemolysis after 48-h incubation. T, total hemolysis.

^*e*^ Reduction of triphenyl 2,3,5 tetrazolium (TTC) to formazan and adsorption of bromothymol blue on NBTA medium after 48-h incubation. Blue colonies: TTC degradation and BBT adsorption. Red colonies: TTC degradation.

### PAX peptides are involved in biofilm formation and swimming motility

**Fig 3:**
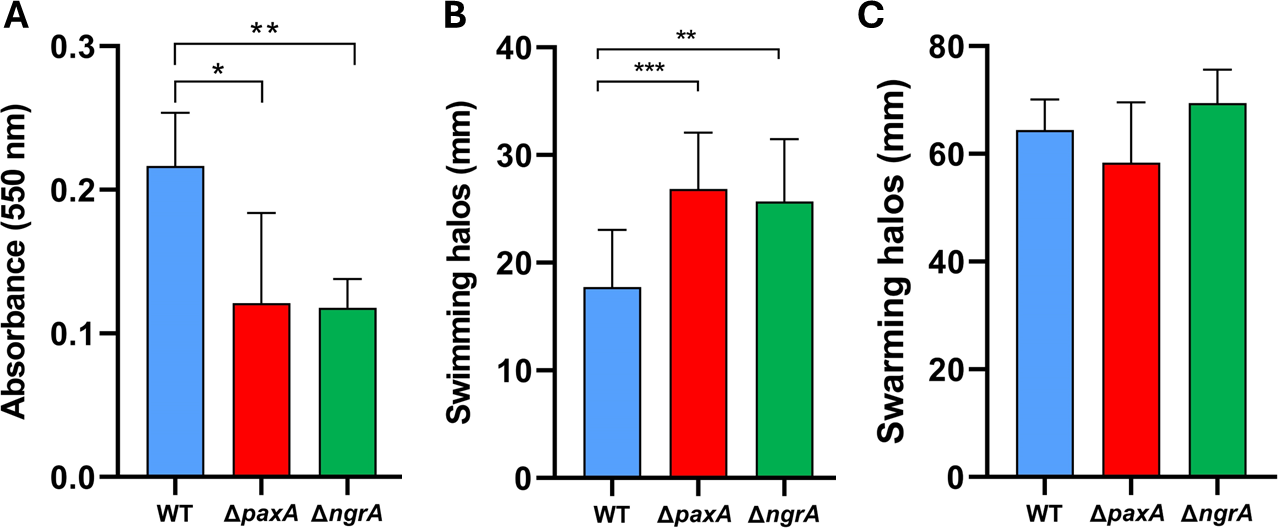
Biofilm formation, swimming motility, and swarming motility of *X. nematophila* WT (blue), ∆*paxA* (red), and ∆*ngrA* (green). (**A**) Biofilm formation was assessed by 1% crystal violet staining of 48 h static bacterial cultures and quantification of absorbance at 550 nm. Student’s *t* test, *n* = 4. Swimming motility (**B**) and swarming motility (**C**) were assessed after dropping 5 µL of overnight bacterial culture onto 0.35% LB agar (Student’s *t* test, *n* = 4) and 0.7% LB agar Eiken (Wilcoxon-Mann-Whitney test, *n* = 4) media, respectively. Halo diameter was measured after 18 h of growth. *P < 0.05, **P < 0.01, and ***P < 0.001.

Biofilm formation, swimming motility, and swarming motility were investigated *in vitro* for *X. nematophila* WT, Δ*paxA,* and Δ*ngrA* strains (Fig. 3). The Δ*paxA* (*P* < 0.05) and Δ*ngrA* (*P* < 0.01) mutants showed significantly lower biofilm formation than the WT strain (Fig. 3A). Conversely, Δ*paxA* (*P* < 0.001) and Δ*ngrA* (*P* < 0.01) mutants showed significantly enhanced swimming motility on 0.35% LB agar medium compared with the WT strain (Fig. 3B). No difference in swarming motility was observed between the three strains on a 0.7% Eiken agar plate (Fig. 3C). PAX peptides are therefore positively involved in biofilm formation and negatively in swimming motility.

### PAX peptides are slightly involved in bacterial virulence toward insects

**Fig 4:**
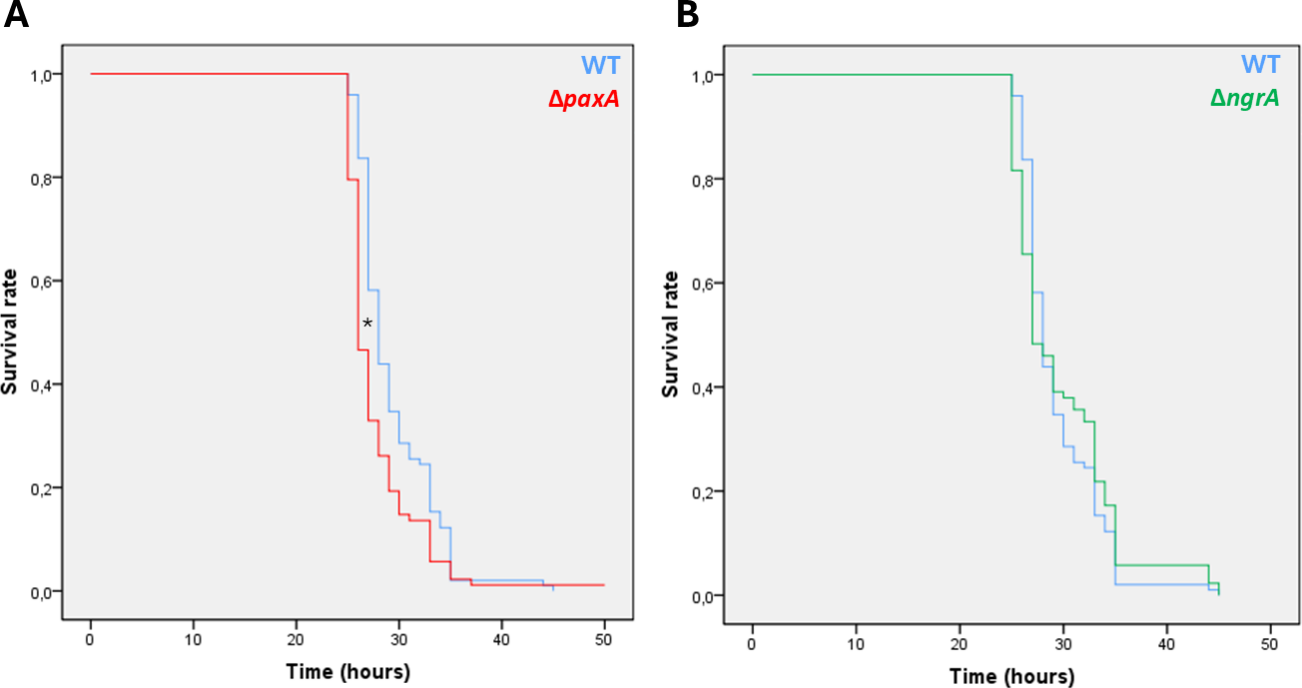
Pathogenicity assay of *X. nematophila* WT (blue), ∆*paxA* (**A**, red), and ∆*ngrA* (**B**, green) toward *S. littoralis* larvae (L6 stage). Kaplan-Meier survival curves were generated with SPSS version 18.0. Each insect was injected with 1,000–3,000 CFUs of the respective strains. Wilcoxon-Mann-Whitney tests, *n* = 80, *P < 0.05.

To determine whether PAX peptides were involved in the virulence of *X. nematophila in vivo*, pathogenicity assays of *X. nematophila* WT, Δ*paxA,* and Δ*ngrA* strains were carried out by injection into *S. littoralis* larvae (Fig. 4). A weak difference (1h40, *P* < 0.05) of larval mortality between the Δ*paxA* mutant and the WT strain was observed (Fig. 4A), but no difference between the Δ*ngrA* mutant and the WT strain (Fig. 4B). In a control experiment, we showed that the WT and mutant strains displayed no growth difference when cultivated in LB (Fig. S2).

To test the hypothesis of a role for PAX peptides in the resistance of *X. nematophila* to insect AMPs (36), MIC assays were carried out using various insect AMPs (cecropin A, cecropin B1, cecropin C, cecropin P1, and drosocin) against the WT, Δ*paxA,* and Δ*ngrA* strains. MIC assays were also carried out using bacterial AMPs (colistin, polymyxin B, NOSO 95C, synthetic PAX1′, synthetic PAX2′, and synthetic PAX7) to determine whether PAX peptides would also enable resistance to competitive bacteria within the insect cadaver (Table 2; Fig. S1). No clear difference in MIC was detected between the WT and mutant strains for any of the bacterial or insect AMPs tested. In our experiments, PAX peptides were not involved in the resistance of *X. nematophila* to the tested insect and bacterial AMPs.

**TABLE 2:** Minimum inhibitory concentration (µg mL^−1^) assays of insect and bacterial antimicrobial peptides on *X. nematophila* WT, ∆*paxA,* and ∆*ngrA*

Antimicrobial peptide	WT	∆*paxA*	∆*ngrA*
Cecropin A	12.5	12.5	10.5
Cecropin B1	6.25	6.25	6.25
Cecropin C	12.5	12.5	25
Cecropin P1	>12.5	>12.5	12.5
Drosocin	>50	>50	>50
Colistin	3.9	1.95	3.9
Polymyxin B	0.245	0.245	0.245
NOSO 95C (odilorhabdin)	>1,700	>1,700	>1,700
Synthetic PAX1′	>1,500	>1,500	>1,500
Synthetic PAX2′	187.5	375	375
Synthetic PAX7	187.5	187.5	375

### Microorganisms inhabiting the ecological niche of *Xenorhabdus* exhibit low susceptibility to PAX peptides

**Fig 5:**
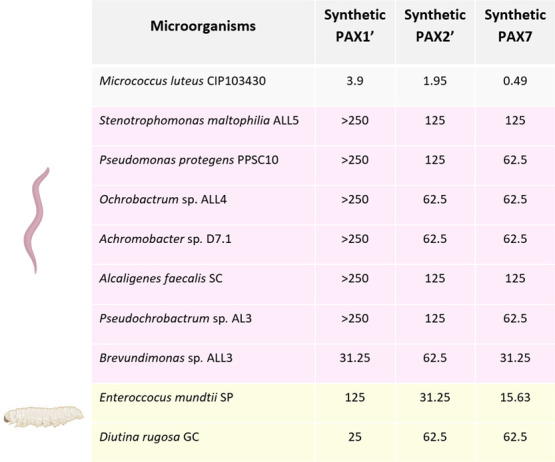
Minimum inhibitory concentration (µg mL^−1^) assays of synthetic PAX1′, PAX2′, and PAX7 on selected microorganisms from nematode microbiota (*Steinernema carpocapsae*, pink) and insect microbiota (*Spodoptera* larvae, yellow).

To determine whether the production of PAX peptides could enable *Xenorhabdus* to outcompete the microorganisms present in the insect cadaver, MIC assays were performed using synthetic PAX1′, PAX2′, and PAX7 (Fig. 5; Fig. S1). For this purpose, 10 microorganisms from the microbiota (FAM) of *S. carpocapsae* nematodes (*Stenotrophomonas maltophilia* ALL5, *Pseudomonas protegens* PPSC10, *Ochrobactrum* sp. ALL4, *Achromobacter* sp. D7.1, *Alcaligenes faecalis* SC, *Pseudochrobactrum* sp. AL3, and *Brevundimonas* sp. ALL3) and from the microbiota of the insect *Spodoptera* (*Enterococcus mundtii* SP and *Diutina rugosa* GC) were selected. *M. luteus* CIP103430 was also used as a positive control for the antimicrobial activity of synthetic PAX peptides, as it had already been identified as susceptible to PAX peptides (28). Microorganisms from the nematode microbiota appear to exhibit low susceptibility to synthetic PAX tested with a minimum MIC of 31.25 µg mL^−1^ for PAX1′ and PAX7 against *Brevundimonas* sp. *E. mundtii* and *D. rugosa* (insect microbiota) show higher susceptibilities, with MICs down to 15.63 µg mL^−1^ for PAX7 against *E. mundtii* and 25 µg mL^−1^ for PAX1′ against *D. rugosa*. The antimicrobial effect of synthetic PAX peptides against the microorganisms co-occurring with *Xenorhabdus* in the insect cadaver niche is moderate.

### PAX peptides enhance infective juvenile nematode production

**Fig 6:**
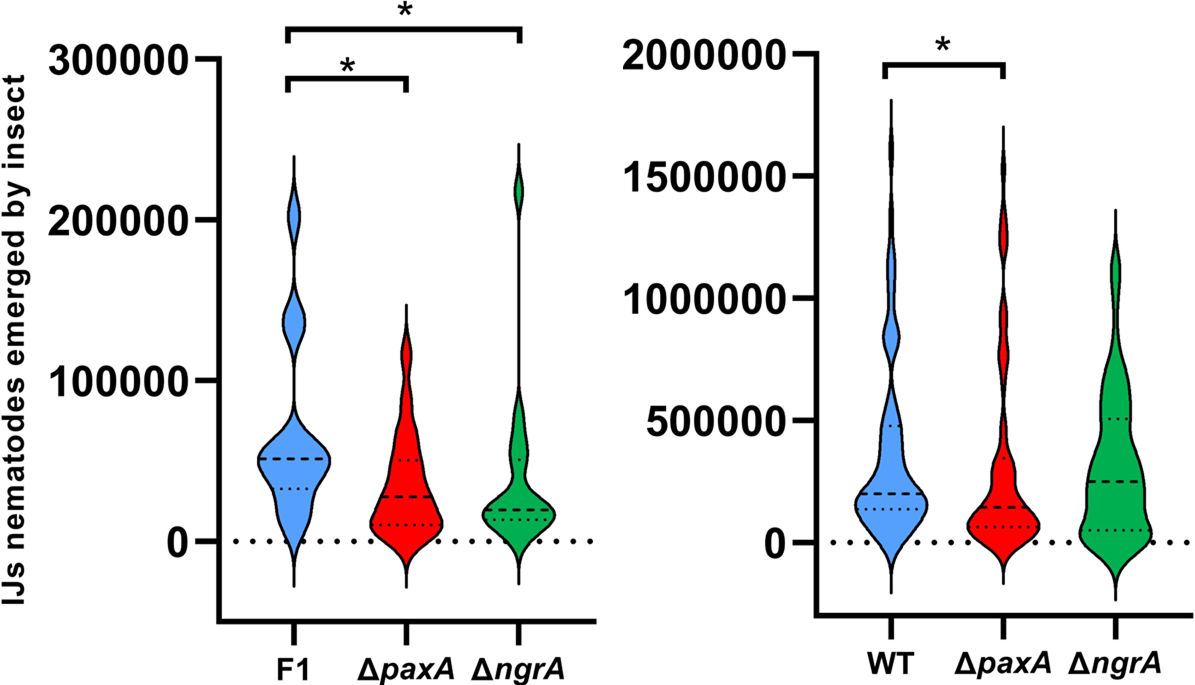
Reproductive success of aposymbiotic *Steinernema carpocapsae* SK27 nematodes reassociated with *X. nematophila* WT, ∆*paxA,* or ∆*ngrA* in *G. mellonella* larvae. Emerging IJ nematodes were counted at 15 and 30 days post-mortem and cumulated. The total number of IJs emerging per insect is represented at the end of the first generation (*n* = 14–19) (left) and second generation (*n* = 60) on three independent experiments (right). Wilcoxon-Mann-Whitney test, *P < 0.05.

Since PAX peptides were detected during the necrotrophic stage (Fig. 1), we questioned whether PAX peptides are involved in the production of IJ nematodes. *G. mellonella* larvae were infested with *S. carpocapsae* SK27 reassociated with the WT strain, Δ*paxA,* or Δ*ngrA* mutants (Fig. 6). A difference in reproductive success was observed between the WT strain and both mutant strains (*n* = 14–19) at the end of the first generation (Fig. 6, left), then between the WT strain and the Δ*paxA* mutant (*n* = 60) at the end of the second generation (Fig. 6, right, Wilcoxon-Mann-Whitney tests, *P* < 0.05). Additionally, no significant difference in the timing of IJ emergence was noticed in both mutants (Fig. S4). The second generation selects the best-performing IJs capable of completing a full life cycle, thereby reducing the differences observed between strains. Furthermore, no significant differences in CFU per IJs were detected at the second generation between the WT strain and both mutant strains (Fig. S6). PAX peptides are therefore involved in the production of *S. carpocapsae* IJs *in vivo*.

### PAX peptide-producing ability is widespread within the *Xenorhabdus* genus

**Fig 7:**
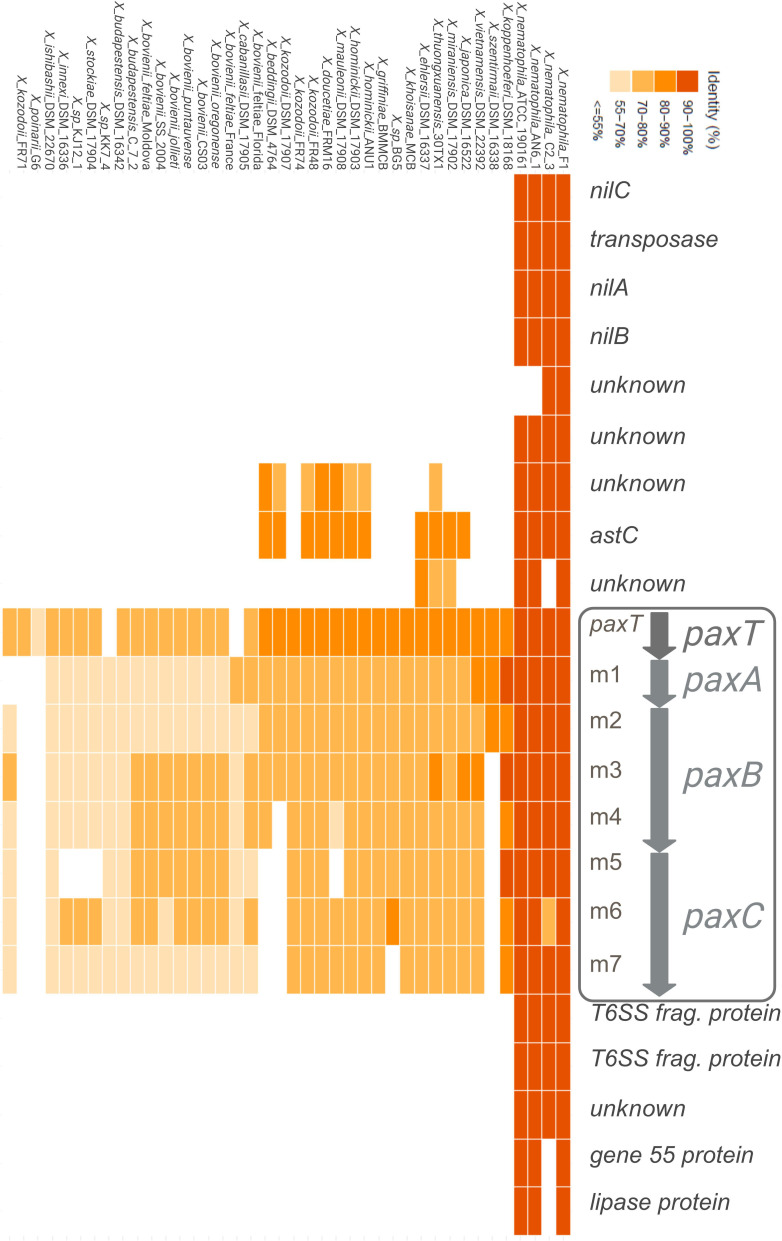
Distribution and genetic environment of *paxTABC* among the *Xenorhabdus* genus. The *paxTABC* and surrounding genes of *X. nematophila* F1 were aligned with those of 40 strains belonging to 23 *Xenorhabdus* species by NCBI BLAST+blastn. The NRPS genes within this cluster were specifically analyzed by comparing their module nucleotide sequences (*paxA*: m1; *paxB*: m2, m3, and m4; *paxC*: m5, m6, and m7). Orange gradient squares represent the nucleotide percentage identity of genes and modules for each orthologous sequence compared to their counterpart of *X. nematophila* F1. The *paxTABC* operon is shown at the top of the figure, along with its surrounding genes in *X. nematophila* F1 genome.

To determine whether PAX peptide production ability is conserved within the *Xenorhabdus* genus, we first studied the distribution and the genetic environment of the *paxTABC* genes in 40 strains of *Xenorhabdus* belonging to 23 species compared to *X. nematophila* F1 (Fig. 7). The surrounding genes and their organization are conserved among *X. nematophila* species, especially the *nilABC* genes, but not in other *Xenorhabdus* species. For NRPS genes, modules (*paxA* [m1], *paxB* [m2, m3, and m4], and *paxC* [m5, m6, and m7]) were analyzed independently as functional entities. *paxTABC* genes are present in almost all studied *Xenorhabdus* strains, except *Xenorhabdus poinarii* G6 and *Xenorhabdus ishibashii* DSM 22670. Although the entire cluster is well distributed among the *Xenorhabdus* strains, the *paxC* gene (m5, m6, and m7) is absent in a few genomes (*Xenorhabdus szentirmaii* DSM 16338, *Xenorhabdus kozodoii* DSM 17907, and *Xenorhabdus beddingii* DSM 4764). Pairwise alignments showed that the *paxTABC* cluster exhibits high conservation across all strains within *Xenorhabdus bovienii* species and within *X. nematophila* species but shows lower identity between the two species (Fig. S5). Thus, the *paxTABC* cluster is present throughout the entire *Xenorhabdus* genus, but with variability among NRPS modules.

**Fig 8:**
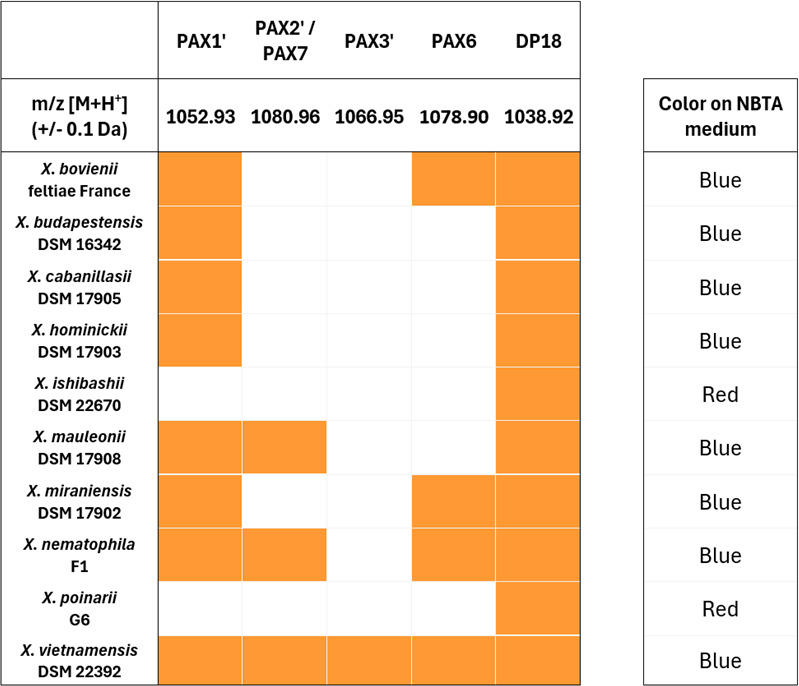
MALDI-TOF-MS detection of selected PAX peptide production in extracts from different strains of *Xenorhabdus* and colony color on NBTA medium. Orange squares indicate that the *m/z* value of the corresponding PAX peptide was detected at ±0.1 Da. White squares mean that no *m/z* value corresponding to PAX peptides was detected. A synthetic PAX peptide derivative DP18 (*m/z* = 1,038.92 Da) was added as a positive extraction control before the extraction steps. The corresponding mass spectra are given in Table S3. Colony colors were assessed after streaking on NBTA medium and incubating for 48 h at 28°C.

To correlate the presence of the *paxABC* genes with the actual production of PAX peptides, mass spectrometry analysis (MALDI-TOF-MS) was then performed on extracts from 48-h cultures of nine different strains representative of the main species of *Xenorhabdus* (Fig. 8; Table S3). The analysis focused on the *m/z* values (±0.1 Da) of the most detectable PAX peptides, PAX1′, PAX2′/PAX7, PAX3′, PAX6, and DP18 as the positive extraction control. The DP18 control was detected in all samples. At least one of the PAX peptides was detected in all tested strains, except *X. poinarii* G6 and *X. ishibashii* DSM 22670 (Table S3), for which no *paxTABC* cluster was evidenced (Fig. 6). The most conserved PAX peptide in all producing strains is PAX1′. These results revealed that the PAX peptide-producing ability is widespread in the *Xenorhabdus* genus.

To determine whether a correlation could be established between BBT adsorption and actual PAX peptide production, we examined the color of colonies grown on NBTA medium (Fig. 8). Similarly to the ∆*paxA* and ∆*ngrA* mutants (Fig. S3), colonies of *X. poinarii* G6 and *X. ishibashii* DSM 22670, lacking the *paxTABC* cluster and for which no PAX peptides were detected in liquid culture, were red on NBTA medium. Therefore, the *Xenorhabdus* strains for which PAX peptide production was demonstrated were systematically observed as blue colonies on NBTA medium, whereas red colonies corresponded to strains unable to produce PAX peptides.

## DISCUSSION

### BBT dye adsorption by bacterial colonies is a marker of PAX peptide production

PAX peptides constitute a family of positively charged cyclolipopeptides, presumably localized at the surface of *Xenorhabdus* bacteria (36). To investigate the role of PAX peptides, Δ*paxA* and Δ*ngrA* mutants were used. Mass spectrometry analysis confirmed the absence of PAX peptide production in both mutants, demonstrating that PAX peptide production in *Xenorhabdus* is *ngrA*-dependent. The NgrA PPTase is therefore likely involved in activation of the PaxABC synthetase, as well as other synthetases responsible for the production of NRP metabolites and optimal antibiotic activities in *Xenorhabdus* (11, 12, 38).

Phenotypic characterization of laboratory cultures revealed similar defects in both Δ*paxA* and Δ*ngrA* mutant strains. This suggests that the changes observed in the NRPS-deficient Δ*ngrA* mutant, including the absence of lecithinase-like activity, increased Tween-lipase activity, abolition of BBT adsorption, reduced biofilm formation, and increased swimming motility, are primarily due to the absence of PAX peptides. We showed that both mutants exhibited an enhanced precipitation zone in the agar containing Tween 20, 40, 60, or 80 and abolished lecithinase-like activity. Initially, lecithinase-like activity in *X. nematophila* F1 was partially characterized through the purification of five compounds that cause lecithin precipitates on agar, without exhibiting phospholipase C activity (39). Given the absence of lecithin precipitates in the Δ*paxA* mutant, it is likely that these five compounds correspond to the five PAX peptides described by (28), who employed the same methodology.

Additionally, our study uncovered another striking difference between both mutants and the WT strain, as colonies of both mutants displayed no adsorption of bromothymol blue dye on NBTA medium, unlike the WT strain. Historically, the phenotype of secondary variants has been used to distinguish variants in *Xenorhabdus*. Indeed, the WT strain shows blue colonies on NBTA medium, while secondary variants exhibit red colonies (40). Initially, this phenomenon was referred to as “phase variation.” Further characterization of *X. nematophila* secondary variants revealed additional phenotypic traits, such as reduced motility, antibiotic activity, hemolytic activity, and lipolytic activities (41–43). Volgyi et al. proposed the term “phenotypic variation” to more accurately describe this phenomenon (44). Recent studies have shown that secondary variants display a growth advantage in stationary phase both *in vitro* and in insects but are less efficiently transmitted by the nematode than the WT strain (43). The switch to secondary forms in *Xenorhabdus* has been linked to mutations in the *lrp* gene (43), and the expression of the *paxAB* gene is known to be Lrp-dependent (45). Indeed, an *lrp* mutant exhibits all the phenotypic traits of a secondary variant, whereas the Δ*paxA* mutant shares only some of them, including BBT adsorption, lecithinase-like activity, and abolished PAX production (46). Moreover, Lrp is a global activator that affects the expression of NRPS genes in *Xenorhabdus* (47). Our mass spectrometry analysis confirmed that *Xenorhabdus* strains that adsorbed BBT on NBTA medium were able to produce PAX peptides in culture. Conversely, *X. poinarii* G6, *X. ishibashii* DSM 22670, and secondary variants form red colonies on NBTA medium, exhibit lecithinase-negative phenotypes (40), and do not produce PAX peptides in culture (Table S3).

Here, we demonstrated that PAX peptides were related to phenotypes involved in phenotypic variation in *Xenorhabdus* and that the blue phenotype on NBTA medium is a reliable marker for PAX peptide production.

### PAX peptides display multiple roles throughout the life cycle of *Xenorhabdus*

While specialized metabolites produced by soil bacteria have been widely investigated in laboratory conditions for their potential use as antimicrobials in diverse applications, only a few studies have addressed their natural roles, and little is known about their effective occurrence in natural environments and/or in mutualistic relationships with hosts (48–50). Pioneer *in vivo* studies on *Xenorhabdus* specialized metabolites demonstrated antimicrobial activity partially attributed to the presence of xenocoumacin in *G. mellonella* larvae infected with *X. nematophila*, with detectable levels persisting for up to 10 days post-infection (51).

Similarly, we monitored the presence of *Xenorhabdus* PAX peptides in the insect cadavers through mass spectrometry analysis in a dynamic way over the course of its natural life cycle. We showed that under simple *in vitro* conditions, PAX peptides were detected from 20 h to 7 days. Under conditions aiming to reproduce the natural life cycle of *X. nematophila* in the insect cadaver (*G. mellonella* infected by *X. nematophila* or infested by *S. carpocapsae*), PAX peptides were detected quite uniformly from 48 h to 7 days post-infection/infestation, but higher variability was observed at 20 h between biological samples. Therefore, PAX peptides are present under natural conditions over a long period of the *Xenorhabdus* life cycle: from the pathogenic phase to the late necrotrophic phase, suggesting that PAX peptides might be involved in various biological functions in *Xenorhabdus*.

PAX peptides may be involved in crucial phenotypes that enable the bacteria to successfully complete the different steps of their infectious cycle, such as bacterial pathogenicity. In our pathogenicity assays using *S. littoralis*, we observed only minor differences between the Δ*paxA* mutant and the WT strain. Contrary to our observations, delayed mortality of *Manduca sexta* larvae had previously been observed when infected with a *ngrA* mutant of *X. szentirmaii* (38). Moreover, the authors have shown that *X. szentirmaii* produces specific *ngrA*-dependent compounds that are absent in *X. nematophila* and could be involved in virulence (38). Previous studies suggested that PAX peptides may protect *Xenorhabdus* against the humoral immune response of insects, including AMPs. Because AMPs are cationic peptides, PAX peptides may induce repulsive force due to positive charge at the bacterial cell wall (36). However, we showed that the MICs of several insect AMPs were similar for the WT and Δ*paxA* or Δ*ngrA* mutants. The observed differences between our study and previous work (36) can be attributed to variations in bacterial species. Therefore, the potential role of PAX peptides during the pathogenic stage could not be inferred from these experiments.

PAX peptides were originally described as antimicrobial peptides (28); thus, they might play a role in inhibiting the growth of competing microorganisms introduced in the insect hemocoel upon infection by IJs (6, 7), or within the cadaver during the necrotrophic stage. However, we showed that nematode microbiota (FAM) (7) and insect microbiota displayed low susceptibilities to the three synthetic PAXs tested (PAX1′, PAX2′, and PAX7). PAX peptides, therefore, do not appear to be required to outcompete microbial competitors.

PAX peptides could be detected up to 7 days post-infection/infestation in our *in vivo* experiment (Fig. 1), which is the period when nematodes begin to emerge from the insect cadaver as IJs (Fig. S4). Nematode reproductive success assays in *G. mellonella* revealed significantly lower progeny using the Δ*paxA* and Δ*ngrA* mutants compared to the WT strain, demonstrating that PAX peptides are involved in the production of IJs. Previous studies had shown that *ngrA*-dependent compounds were required for the development of the nematode partner in two different nematode genera: *H. bacteriophora* associated with *P. luminescens* and *S. carpocapsae* associated with *X. nematophila* AN6 (12, 13). Our result strongly suggests that PAX peptides are the *ngrA-*dependent metabolites necessary for full IJ production in the *X. nematophila-S. carpocapsae* mutualism.

As cyclolipopeptides are often identified as involved in biofilm formation, swimming motility, and swarming motility (50), we assessed the involvement of PAX peptides in these phenotypes. Indeed, these features could play a role in successive bacterial colonization of the nematodes in the insect cadaver, prior to IJ emergence. We have highlighted that PAX peptides are positively involved in biofilm formation and negatively involved in swimming motility, but no difference was found in swarming motility. It has been shown that *X. nematophila* can produce biofilms to adhere to the heads of *C. elegans* nematodes, while secondary variants that are unable to produce PAX peptides or a biofilm-deficient mutant lack this ability (52). Another study that conducted experimental evolution involving *Pseudomonas lurida* and *C. elegans* showed that biofilm formation was a bacterial trait associated with a mutualistic lifestyle and motility associated with a free-living lifestyle (53). In addition, *P. lurida* produces cyclolipopeptides involved in a mutualistic relationship with the nematode *C. elegans* (54). We can presume that PAX peptides, which promote biofilm formation, could help the bacterium to associate with its nematode. Other studies revealed that *C. elegans* nematodes could directly sense cyclolipopeptides (serrawettin W2) produced by harmful bacteria (S*erratia marcescens*) with their chemosensory neurons, which had a repulsive effect on the nematodes (55). It could be hypothesized that PAX cyclolipopeptides may be sensed by the nematode partner to promote mutualism.

All together, these results revealed that PAX peptides are involved in biofilm formation, swimming motility, and the production of nematode IJs.

### The PAX peptide-producing ability is widespread in the genus *Xenorhabdus*

We highlighted that the *paxTABC* cluster is present throughout all the *Xenorhabdus* genus but is restricted to that genus only. A distinct genomic environment around the *paxTABC* cluster was observed among different *Xenorhabdus* species, and a high degree of variability in the percentage of identity within the NRPS gene sequences (*paxABC*) was noticed when blasted to the *X. nematophila* F1 genome. This variability, due to the high rate of recombination, is characteristic of NRPS genes, unlike genes encoding proteins via the ribosomal pathway (10, 56, 57). We therefore performed the NRPS gene analysis on the modules rather than on the whole genes. Despite the NRPS module’s sequence variability, the seven different strains representative of the main *Xenorhabdus* species all produced PAX1′ (lysine in position 2), while PAX peptides with an arginine in position 2 (PAX2′ or PAX6) were also detectable in a few strains. In addition to our analysis, the production of PAX peptides has already been confirmed in eight other strains, including *X. nematophila* ATCC 19061, *Xenorhabdus stockiae* DSM 17904, *Xenorhabdus* sp. KJ12.1, *Xenorhabdus* sp. PB62.4, *X. doucetiae* FRM16, *Xenorhabdus khoisanae* SB10, *X. khoisanae* J194 and *X. doucetiae* DSM 17909 (9, 32, 33, 36, 58) . Nevertheless, it is important to note that not all PAX peptides are detected in all the analyzed strains, and the only PAX peptide consistently detected is PAX1′. The ability to produce PAX peptides is therefore both widespread and unique to the *Xenorhabdus* genus.

The only non-PAX-producing species among those tested are *X. ishibashii* and X. *poinarii. X. poinarii* has the particularity of being defective in the production of IJs of its nematode partner *Steinernema glaseri*, being avirulent without its nematode and showing strong genomic reduction (40, 59–61). Moreover, a large proportion of *S. glaseri* nematodes are naturally aposymbiotic (40). It is conceivable that the absence of PAX peptide production by *X. poinarii* may contribute to its inefficient association with its nematode partner. Furthermore, in *X. nematophila,* the *paxTABC* genes are clustered close to the *nilABC* genes, whose proteins have been shown to be involved in species-specific colonization of *S. carpocapsae* (62–65). The *nilABC* cluster is restricted to the *X. nematophila* species, whereas the *paxTABC* genes are present throughout the whole *Xenorhabdus* genus. We can therefore assume that the PAX peptides unique to the *Xenorhabdus* genus are metabolites involved in the mutualistic association between the bacterium and its nematode host.

Finally, PAX peptides unique to the *Xenorhabdus* genus are metabolites involved in many functions such as swimming motility, biofilm formation, and the production of infective juvenile nematodes, suggesting their involvement in the interaction between the bacterium and its nematode host.

## MATERIALS AND METHODS

### Bacterial strains, plasmids, and growth conditions

The strains and plasmids used in this study are listed in Fig. S4. Bacteria were routinely grown in Lysogeny Broth (Sigma-Aldrich) medium at 28°C (*Xenorhabdus, S. maltophilia, P. protegens, Ochrobactrum* sp*., Achromobacter* sp*., A. faecalis, Pseudochrobactrum* sp*., Brevundimonas* sp*., E. mundtii,* and *D. rugosa*) or 37°C (*Escherichia coli* and *M. luteus*). When required, antibiotics were used at the following final concentrations: gentamicin (Sigma-Aldrich), 20 µg mL^−1^ and chloramphenicol (Sigma-Aldrich), 20 µg mL^−1^ for *E. coli* strains and 15 µg mL^−1^ for *X. nematophila* (or 8 µg mL^−1^ for allelic exchange).

### Molecular genetic techniques

DNA manipulations were performed as previously described (66). Plasmids were introduced into *E. coli* by transformation and transferred to *X. nematophila* by conjugative mating with WM3064 as donor strain (67). All constructs were sequenced by Eurofins Genomics Germany GmbH. The primers used in this study (IDT) are described in Table S5.

### Construction of *X. nematophila* Δ*paxA* mutant

The Δ*paxA* mutant was constructed using the same experimental strategy as for the construction of the Δ*ngrA* mutant from the WT strain *X. nematophila* F1 (37). The upstream and downstream regions of the *paxA* gene were amplified by PCR with the L-PCR1-*paxA*-SalI and R-PCR1-*paxA*-BamHI primers for the upstream region (621 bp) and the L-PCR2-*paxA*-BamHI and R-PCR2-*paxA*-SpeI primers for the downstream region (601 bp). The two fragments obtained were inserted, together with the 3.8 kb *BamHI* fragment ΩCam cassette from pHP45-ΩCm, conferring resistance to chloramphenicol, into pJQ200SK digested with *SalI* and *SpeI* for insertion of the ΩCam cassette between the two PCR fragments. The resulting plasmid, pJQ-*paxA*::ΩCm, was used to transform *E. coli* WM3064 and was introduced into *X. nematophila* F1 by mating. Allelic exchange was performed as previously described (68). ΩCam insertion was confirmed by PCR analysis using primers L-*pax*TA and R-verif-*pax*. The absence of PAX peptide products was assessed by mass spectrometry analysis, as described below. The clone obtained was called Δ*paxA.*

### Phenotypic assays

Phenotypic characteristics of Δ*paxA* and Δ*ngrA* mutants were compared to those of the WT strain by streaking or dropping 5 µL of overnight preculture on the following agar media, as previously described by Boemare et al.: NBTA (Difco Nutrient Agar 1.5% [BD]) supplemented with 25 mg L^−1^ Bromothymol blue (Merck Millipore) and 40 mg L^−1^ triphenyl 2,3,5 tetrazolium (Sigma-Aldrich) (41); Difco Nutrient Agar 1.5% supplemented with 0.1 g L^−1^ CaCl_2_ and 1% Tween 20 (polyoxyethylene sorbitan monolaurate), Tween 40 (polyoxyethylene sorbitan monopalmitate), Tween 60 (polyoxyethylene sorbitan monostearate), Tween 80 (polyoxyethylene sorbitan monooleate), or Tween 85 (polyoxyethylene sorbitan monotrioleate) (Sigma-Aldrich); Difco Nutrient Agar 1.5% supplemented with 1% egg lecithin (VWR Avantor); Trypticase Soy Agar (BioMerieux) supplemented with 7% sheep blood (Eurobio Scientific) (69); Difco Nutrient Agar supplemented with a 6 g L^−1^ soft agar overlay containing 2% *M. luteus* preculture; 0.35% LB Agar medium for swimming motility assays (70); and LB supplemented with 0.7% of Eiken Agar (Gerbu) for swarming motility assays (71).

### Biofilm assays

Crystal violet biofilm assays were carried out following the methods described by Pothula et al., using LB broth instead of LPB medium (72). Briefly, cultures grown for 48 h without agitation were stained with 1% crystal violet (Sigma-Aldrich), then dissolved in 30% acetic acid after washing steps. The absorbance of samples in a 96-well plate was quantified at 550 nm in a TECAN Infinite 200 plate reader. Each experiment was conducted with four independent clones and repeated four times for each mutant strain. Data were analyzed using GraphPad Prism 9 software, and significance was tested using Student’s test.

### MIC determination

MICs were determined in accordance with CLSI guidelines (73) with the following modifications: 5 mL of MHB broth (Bio-Rad) was inoculated with an overnight culture in LB broth and grown to a 0.6 < OD_600_ < 0.9. The inoculum for the microplate was prepared as described in the CLSI guide, with dilution in MHB to approximately 10^4^ CFU mL^−1^. Ninety-six-well low-binding plates were incubated for 48 h at 28°C with slow shaking. The peptides used in the analyses were supplied by the following providers: cecropin A (Sigma-Aldrich), cecropin B1 (NeoMPS S.A.) (74), cecropin C (Proteogenix), cecropin P1 (Sigma-Aldrich), drosocin (Eurogentec), colistin (Sigma-Aldrich), polymyxin B (Sigma-Aldrich), and NOSO 95C (odilorhabdin, Nosopharm S.A. [16]). Synthetic PAX1′, PAX2′, and PAX7 were provided by the SynBio3 platform (IBMM, Montpellier, France), and their structure is shown in Fig. S1.

### Pathogenicity assays

Bacterial pathogenicity was assessed by injecting *Xenorhabdus* into *S. littoralis*, as previously described (75). Briefly, bacterial cultures in LB broth (OD_600_ ≈ 0.8) were diluted in the culture medium, and 20 µL of the resulting bacterial suspension, containing 1,000–3,000 CFUs, was injected into the hemolymph of 20 sixth-instar larvae of *S. littoralis*. The number of bacterial cells injected into the larvae was determined by plating on nutrient agar and counting the CFUs. After the bacterial injection, the insect larvae were incubated at 23°C, and mortalities were monitored for up to 50 h. The pathogenicity of bacterial isolates was determined by measuring the time required for 50% of the insect larvae to be killed (LT_50_). The results of four independent experiments were combined and analyzed with the software Statistical Package for the Social Sciences version 18.0. Significant differences between two data sets were assessed with non-parametric Wilcoxon-Mann-Whitney tests, with a 95% confidence interval.

### Nematode colonization assays

Bacterium–nematode complexes were produced using an *in vivo* technique (2, 76) in which *G. mellonella* (wax moth) larvae were infected with *X. nematophila* F1 strain and aposymbiotic IJs of *S. carpocapsae* (SK27, Plougastel) nematodes. Aposymbiotic nematodes were obtained previously (77). Approximately 100 aposymbiotic IJs per larvae were first placed on a piece of filter paper in individual Eppendorf tubes and incubated with 20 larvae of *G. mellonella* at 23°C. After 24 h of infestation, insect larvae were injected with 20 µL (~3,000 bacteria) of WT, Δ*paxA,* or Δ*ngrA* strains that had been grown until OD_600_ ≈ 0.8. After insect death (24–48 h post-infestation), cadavers were transferred to White traps (78). Fourteen and thirty days after infestation, the IJs emerging from the cadavers were collected in Ringer’s solution (Aguettant). Once again, these IJs were then used to infest new *G. mellonella larvae*. Nematodes were collected and washed with water through a 20 µm filter and stored in Ringer’s solution at 9°C in tissue culture flasks. Reproductive success (number of IJ nematodes emerging from an insect larva) was determined by cumulating the number of nematodes collected and counted at 15 and 30 days post-infestation. Direct counts of average CFU/nematode were determined via a grinding assay, wherein collected nematodes were equalized by density, serially diluted, and plated on NBTA plates (Fig. S6). Each experiment was repeated three times. Data were analyzed using GraphPad Prism 9 software, and significance was tested using non-parametric Wilcoxon-Mann-Whitney tests.

### Mass spectrometry analysis

#### PAX peptide extraction from *Xenorhabdus* cultures

PAX extraction methods were adapted from those previously described by (28, 32, 36). Tubes of 5 mL LB were inoculated with 200 µL preculture of overnight *Xenorhabdus* strains and grown for 48 h at 28°C under agitation. Cultures were centrifuged at 6,000 × *g* for 10 min, and the supernatant fraction was discarded. The cell pellet fraction was resuspended in 1 mL LB, and 45 µg of DP18 (R-3-Hydroxytetradecanoic acid-Gly-Orn-[DLys-Lys-DLys-DLys-Lys], *m/z* = 1,038.92 Da), a synthetic PAX peptide derivative, was added as an extraction control. Samples were sonicated for 2 min, then centrifuged at 16,000 × *g* for 5 min. The supernatant corresponding to the cytosolic fraction (S1) was conserved. The new pellet containing membrane debris was resuspended in 500 µL methanol/H_2_O (vol/vol) and acidified with 1% formic acid. Samples were incubated for 5 min in the ultrasonic bath. The insoluble fractions were then separated by centrifugation (16,000 × *g*, 5 min). The resulting supernatant S2 was pooled with supernatant S1 and then filtered with 0.45 µm filters (Filtropur, Sarstedt). Samples were suspended at a 1:1 ratio in 0.1 M NaCl, 0.02 M Tris, pH 9 (vol/vol), then loaded onto Sep-Pak CarboxyMethyl Short Cartridge (Accell Plus CM, Waters) prepared according to the manufacturer’s instructions. Cartridges were eluted with 0.5 M NaCl, 0.02 M Tris, pH 9, and 0.1% trifluoroacetic acid. The resulting peptide extract was then loaded onto Sep-Pak C18 Short cartridge (Sep-Pak Plus C18, Waters) prepared according to the manufacturer’s instructions. Peptides were eluted with 100% acetonitrile (ACN) and freeze-dried.

#### Preparation of *in vivo* samples

*G. mellonella* larvae were injected with 5 × 10^3^ to 7 × 10^3^ CFUs of *X. nematophila* F1 (as described in Pathogenicity assays) or infested with 100 *S. carpocapsae* SK27 IJ nematodes (as described in Nematode colonization assays) and incubated at 23°C. Larvae were weighed and frozen at −20°C at each time point post-injection/infestation: 20 h, 48 h, 72 h, 5 days, and 7 days. Larvae surfaces were first disinfected with 70% ethanol, after which the heads were removed. Insect forceps were then used to extrude internal contents. The insides of 10 larvae per condition were pooled and added to 2 mL 0.05 M Tris, pH 7, before grinding using FastPrep-24 5G (MP Biomedicals) with sterile glass beads (6.5 m/s, 30 s, three times). Samples were centrifuged at 6,000 × *g* for 30 min, and cell pellets were resuspended in 1.5 mL 0.05 M Tris, pH 7. PAX peptide extraction was then performed as described above, starting from the step of DP18 addition to the samples. Experiments were performed in triplicate.

#### MALDI-TOF-MS analysis

Extracted peptides were analyzed with a RapifleX MALDI TOF/TOF (Bruker) equipped with a Pulse Smart Beam 3D laser at 335 nm. The following instrument parameters were used: frequency, 10,000 Hz; delayed extraction time, 160 ns; source, positive mode; reflectron mode. Samples were resuspended in ACN and mixed at a 1:1 ratio with 1 µL of saturated α-cyano-4-hydroxycinnamic acid. A volume of 1 µL of the mix was spotted onto a stainless-steel target and air-dried. A targeted analysis of the spectra was carried out on compounds with *m/z* ratios between 1,000 and 1,200, and corresponding monoisotopic mass lists were generated using Flex Analysis 3.4 (Bruker).

#### NRPS genomic analysis

The *paxTABC* and surrounding gene sequences of 40 strains among 23 *Xenorhabdus* species were extracted from the MicroScope MaGe database (https://mage.genoscope.cns.fr/microscope/home/index.php). Module sequences of NRPS genes (*paxA*: m1; *paxB*: m2, m3, and m4; *paxC*: m5, m6, and m7) were extracted using the antiSMASH tool implemented in the MicroScope MaGe database. Strains with genomes of poor assembly quality (>350 contigs) were removed from the analysis. A blastn analysis using the NCBI BLAST+ tool implemented on the Galaxy version 2.10.0+ galaxy1 platform (Galaxy Pasteur, https://galaxy.pasteur.fr/) was performed using the sequence of *X. nematophila* F1 as query with a cutoff value of 0.1 and a minimum coverage percentage >30%. Percentage identities have been used to construct Fig. 6 using the ggplot2 package on RStudio version 2024.09.1+394. Details of the data used for these analyses can be found in Tables S6 and S7.

## SUPPLEMENTAL MATERIAL

Supplemental material aem.00760-25-s0001.pdf Figures S1 to S6 and Tables S1 to S5.

Table S6 aem.00760-25-s0002.xlsx Additional information for blastn analysis of Fig. 6, representing the distribution of *paxTABC* genes and their genetic environment among the genus *Xenorhabdus* retrieved from the MaGe database.

Table S7 aem.00760-25-s0003.xlsx Additional information for blastn analysis of Fig. S5, representing the distance matrix of *paxTABC* genes in the genus *Xenorhabdus* from sequences extracted from the MicroScope MaGe database.

ASM does not own the copyrights to Supplemental Material that may be linked to, or accessed through, an article. The authors have granted ASM a non-exclusive, world-wide license to publish the Supplemental Material files. Please contact the corresponding author directly for reuse.

## References

[1] Goodrich-Blair H, Clarke DJ. 2007. Mutualism and pathogenesis in Xenorhabdus and Photorhabdus: two roads to the same destination. Mol Microbiol 64:260–268. doi:10.1111/j.1365-2958.2007.05671.x 17493120

[2] Sicard M, Brugirard-Ricaud K, Pagès S, Lanois A, Boemare NE, Brehélin M, Givaudan A. 2004. Stages of infection during the tripartite interaction between Xenorhabdus nematophila, its nematode vector, and insect hosts. Appl Environ Microbiol 70:6473–6480. doi:10.1128/AEM.70.11.6473-6480.2004 15528508 PMC525208

[3] Stock SP. 2019. Partners in crime: symbiont-assisted resource acquisition in Steinernema entomopathogenic nematodes. Curr Opin Insect Sci 32:22–27. doi:10.1016/j.cois.2018.10.006 31113627

[4] Nguyen KB, Hunt DJ, Mráček Z. 2007. Steinernematidae: species descriptions, p 121–609. In Entomopathogenic Nematodes: Systematics, Phylogeny, and Bacterial Symbionts. Vol. 5. Nematology Monographs and Perspectives. Koninklijke Brill BV, Leiden, Netherlands.

[5] Walsh KT, Webster JM. 2003. Interaction of microbial populations in Steinernema (Steinernematidae, Nematoda) infected Galleria mellonella larvae. J Invertebr Pathol 83:91–96. doi:10.1016/s0022-2011(03)00079-x 12788281

[6] Singh S, Reese JM, Casanova-Torres AM, Goodrich-Blair H, Forst S. 2014. Microbial population dynamics in the hemolymph of Manduca sexta infected with Xenorhabdus nematophila and the entomopathogenic nematode Steinernema carpocapsae. Appl Environ Microbiol 80:4277–4285. doi:10.1128/AEM.00768-14 24814780 PMC4068695

[7] Ogier JC, Pagès S, Frayssinet M, Gaudriault S. 2020. Entomopathogenic nematode-associated microbiota: from monoxenic paradigm to pathobiome. Microbiome 8:25. doi:10.1186/s40168-020-00800-5 32093774 PMC7041241

[8] Lacey LA, Grzywacz D, Shapiro-Ilan DI, Frutos R, Brownbridge M, Goettel MS. 2015. Insect pathogens as biological control agents: Back to the future. J Invertebr Pathol 132:1–41. doi:10.1016/j.jip.2015.07.009 26225455

[9] Tobias NJ, Wolff H, Djahanschiri B, Grundmann F, Kronenwerth M, Shi YM, Simonyi S, Grün P, Shapiro-Ilan D, Pidot SJ, Stinear TP, Ebersberger I, Bode HB. 2017. Natural product diversity associated with the nematode symbionts Photorhabdus and Xenorhabdus. Nat Microbiol 2:1676–1685. doi:10.1038/s41564-017-0039-9 28993611

[10] Shi YM, Hirschmann M, Shi YN, Ahmed S, Abebew D, Tobias NJ, Grün P, Crames JJ, Pöschel L, Kuttenlochner W, Richter C, Herrmann J, Müller R, Thanwisai A, Pidot SJ, Stinear TP, Groll M, Kim Y, Bode HB. 2022. Global analysis of biosynthetic gene clusters reveals conserved and unique natural products in entomopathogenic nematode-symbiotic bacteria. Nat Chem 14:701–712. doi:10.1038/s41557-022-00923-2 35469007 PMC9177418

[11] Beld J, Sonnenschein EC, Vickery CR, Noel JP, Burkart MD. 2014. The phosphopantetheinyl transferases: catalysis of a post-translational modification crucial for life. Nat Prod Rep 31:61–108. doi:10.1039/c3np70054b 24292120 PMC3918677

[12] Singh S, Orr D, Divinagracia E, McGraw J, Dorff K, Forst S. 2015. Role of secondary metabolites in establishment of the mutualistic partnership between Xenorhabdus nematophila and the entomopathogenic nematode Steinernema carpocapsae. Appl Environ Microbiol 81:754–764. doi:10.1128/AEM.02650-14 25398871 PMC4277586

[13] Ciche TA, Bintrim SB, Horswill AR, Ensign JC. 2001. A phosphopantetheinyl transferase homolog is essential for photorhabdus luminescens to support growth and reproduction of the entomopathogenic nematode Heterorhabditis bacteriophora. J Bacteriol 183:3117–3126. doi:10.1128/JB.183.10.3117-3126.2001 11325940 PMC95212

[14] Booysen E, Dicks LMT. 2020. Does the future of antibiotics lie in secondary metabolites produced by Xenorhabdus spp.? a review. Probiotics Antimicrob Proteins 12:1310–1320. doi:10.1007/s12602-020-09688-x 32844362

[15] McInerney BV, Taylor WC, Lacey MJ, Akhurst RJ, Gregson RP. 1991. Biologically active metabolites from Xenorhabdus spp., Part 2. Benzopyran-1-one derivatives with gastroprotective activity. J Nat Prod 54:785–795. doi:10.1021/np50075a006 1955881

[16] Pantel L, Florin T, Dobosz-Bartoszek M, Racine E, Sarciaux M, Serri M, Houard J, Campagne JM, de Figueiredo RM, Midrier C, Gaudriault S, Givaudan A, Lanois A, Forst S, Aumelas A, Cotteaux-Lautard C, Bolla JM, Vingsbo Lundberg C, Huseby DL, Hughes D, Villain-Guillot P, Mankin AS, Polikanov YS, Gualtieri M. 2018. Odilorhabdins, Antibacterial Agents that Cause Miscoding by Binding at a New Ribosomal Site. Mol Cell 70:83–94. doi:10.1016/j.molcel.2018.03.001 29625040

[17] Zhou Q, Grundmann F, Kaiser M, Schiell M, Gaudriault S, Batzer A, Kurz M, Bode HB. 2013. Structure and biosynthesis of xenoamicins from entomopathogenic Xenorhabdus. Chemistry 19:16772–16779. doi:10.1002/chem.201302481 24203528

[18] Lang G, Kalvelage T, Peters A, Wiese J, Imhoff JF. 2008. Linear and cyclic peptides from the entomopathogenic bacterium Xenorhabdus nematophilus. J Nat Prod 71:1074–1077. doi:10.1021/np800053n 18491867

[19] Reimer D, Nollmann FI, Schultz K, Kaiser M, Bode HB. 2014. Xenortide Biosynthesis by Entomopathogenic Xenorhabdus nematophila. J Nat Prod 77:1976–1980. doi:10.1021/np500390b 25080196

[20] Hacker C, Cai X, Kegler C, Zhao L, Weickhmann AK, Wurm JP, Bode HB, Wöhnert J. 2018. Structure-based redesign of docking domain interactions modulates the product spectrum of a rhabdopeptide-synthesizing NRPS. Nat Commun 9:4366. doi:10.1038/s41467-018-06712-1 30341296 PMC6195595

[21] Cai X, Nowak S, Wesche F, Bischoff I, Kaiser M, Fürst R, Bode HB. 2017. Entomopathogenic bacteria use multiple mechanisms for bioactive peptide library design. Nat Chem 9:379–386. doi:10.1038/nchem.2671 28338679

[22] Bode HB, Reimer D, Fuchs SW, Kirchner F, Dauth C, Kegler C, Lorenzen W, Brachmann AO, Grün P. 2012. Determination of the absolute configuration of peptide natural products by using stable isotope labeling and mass spectrometry. Chemistry 18:2342–2348. doi:10.1002/chem.201103479 22266804

[23] Li J, Chen G, Webster JM. 1997. Nematophin, a novel antimicrobial substance produced by Xenorhabdus nematophilus (Enterobactereaceae). Can J Microbiol 43:770–773. doi:10.1139/m97-110 9304787

[24] Grundmann F, Kaiser M, Kurz M, Schiell M, Batzer A, Bode HB. 2013. Structure determination of the bioactive depsipeptide xenobactin from Xenorhabdus sp.768 PB30.3. RSC Adv 3:22072–22077. doi:10.1039/c3ra42660k

[25] Ohlendorf B, Simon S, Wiese J, Imhoff JF. 2011. Szentiamide, an N-formylated cyclic depsipeptide from Xenorhabdus szentirmaii DSM 16338T. Nat Prod Commun 6:1247–1250. 21941889

[26] Fuchs SW, Sachs CC, Kegler C, Nollmann FI, Karas M, Bode HB. 2012. Neutral loss fragmentation pattern based screening for arginine-rich natural products in Xenorhabdus and Photorhabdus. Anal Chem 84:6948–6955. doi:10.1021/ac300372p 22873683

[27] Kronenwerth M, Bozhüyük KAJ, Kahnt AS, Steinhilber D, Gaudriault S, Kaiser M, Bode HB. 2014. Characterisation of taxlllaids A-G; natural products from Xenorhabdus indica. Chemistry 20:17478–17487. doi:10.1002/chem.201403979 25351611

[28] Gualtieri M, Aumelas A, Thaler J-O. 2009. Identification of a new antimicrobial lysine-rich cyclolipopeptide family from Xenorhabdus nematophila. J Antibiot 62:295–302. doi:10.1038/ja.2009.31 19373275

[29] Touray M, Ulug D, Gulsen SH, Cimen H, Hazir C, Bode HB, Hazir S. 2024. Natural products from Xenorhabdus and Photorhabdus show promise as biolarvicides against Aedes albopictus. Pest Manag Sci 80:4231–4242. doi:10.1002/ps.8127 38619291

[30] Incedayi G, Cimen H, Ulug D, Touray M, Bode E, Bode HB, Orenlili Yaylagul E, Hazir S, Cakmak I. 2021. Relative potency of a novel acaricidal compound from Xenorhabdus, a bacterial genus mutualistically associated with entomopathogenic nematodes. Sci Rep 11:11253. doi:10.1038/s41598-021-90726-1 34045620 PMC8159955

[31] Erkoc P, Schmitt M, Ingelfinger R, Bischoff-Kont I, Kopp L, Bode HB, Schiffmann S, Fürst R. 2021. Xenocoumacin 2 reduces protein biosynthesis and inhibits inflammatory and angiogenesis-related processes in endothelial cells. Biomed Pharmacother 140:111765. doi:10.1016/j.biopha.2021.111765 34058438

[32] Fuchs SW, Proschak A, Jaskolla TW, Karas M, Bode HB. 2011. Structure elucidation and biosynthesis of lysine-rich cyclic peptides in Xenorhabdus nematophila. Org Biomol Chem 9:3130–3132. doi:10.1039/c1ob05097d 21423922

[33] Dreyer J, Rautenbach M, Booysen E, van Staden AD, Deane SM, Dicks LMT. 2019. Xenorhabdus khoisanae SB10 produces Lys-rich PAX lipopeptides and a Xenocoumacin in its antimicrobial complex. BMC Microbiol 19:132. doi:10.1186/s12866-019-1503-x 31195965 PMC6567599

[34] Schmidt R, Ulanova D, Wick LY, Bode HB, Garbeva P. 2019. Microbe-driven chemical ecology: past, present and future. ISME J 13:2656–2663. doi:10.1038/s41396-019-0469-x 31289346 PMC6794290

[35] Abebew D, Sayedain FS, Bode E, Bode HB. 2020. Uncovering nematicidal natural products from Xenorhabdus bacteria. J Agric Food Chem 68:13025–13035. doi:10.1021/acs.jafc.0c05297 34981939 PMC8778618

[36] Vo TD, Spahn C, Heilemann M, Bode HB. 2021. Microbial cationic peptides as a natural defense mechanism against insect antimicrobial peptides. ACS Chem Biol 16:447–451. doi:10.1021/acschembio.0c00794 33596038

[37] Lanois-Nouri A, Pantel L, Fu J, Houard J, Ogier JC, Polikanov YS, Racine E, Wang H, Gaudriault S, Givaudan A, Gualtieri M. 2022. The Odilorhabdin antibiotic biosynthetic cluster and acetyltransferase self-resistance locus are niche and species specific. MBio 13:e0282621. doi:10.1128/mbio.02826-21 35012352 PMC8749412

[38] Ciezki K, Wesener S, Jaber D, Mirza S, Forst S. 2019. ngrA-dependent natural products are required for interspecies competition and virulence in the insect pathogenic bacterium Xenorhabdus szentirmaii. Microbiology (Reading) 165:538–553. doi:10.1099/mic.0.000793 30938671

[39] Thaler JO, Duvic B, Givaudan A, Boemare N. 1998. Isolation and entomotoxic properties of the Xenorhabdus nematophilus F1 lecithinase. Appl Environ Microbiol 64:2367–2373. doi:10.1128/AEM.64.7.2367-2373.1998 9647801 PMC106397

[40] Akhurst RJ. 1986. Xenorhabdus nematophilus subsp. poinarii: its interaction with insect pathogenic nematodes. Syst Appl Microbiol 8:142–147. doi:10.1016/S0723-2020(86)80162-X

[41] Boemare N, Thaler JO, Lanois A. 1997. Simple bacteriological tests for phenotypic characterization of Xenorhabdus and Photorhabdus phase variants. Symbiosis 22:167–175.

[42] Ehlers RU, Stoessel S, Wyss U. 1990. The influence of phase variants of Xenorhabdus spp. and Escherichia coli (Enterobacteriaceae) on the propagation of entomopathogenic nematodes of the genera Steinernema and Heterorhabditis. Revue Nématol 13:417–424.

[43] Cambon MC, Parthuisot N, Pagès S, Lanois A, Givaudan A, Ferdy JB. 2019. Selection of Bacterial Mutants in Late Infections: when vector transmission trades off against growth advantage in stationary phase. mBio 10:e01437-19. doi:10.1128/mBio.01437-19 31594811 PMC6786866

[44] Volgyi A, Fodor A, Forst S. 2000. Inactivation of a novel gene produces a phenotypic variant cell and affects the symbiotic behavior of Xenorhabdus nematophilus. Appl Environ Microbiol 66:1622–1628. doi:10.1128/AEM.66.4.1622-1628.2000 10742251 PMC92032

[45] Engel Y, Windhorst C, Lu X, Goodrich-Blair H, Bode HB. 2017. The global regulators Lrp, LeuO, and HexA control secondary metabolism in entomopathogenic bacteria. Front Microbiol 8:209. doi:10.3389/fmicb.2017.00209 28261170 PMC5313471

[46] Cowles KN, Cowles CE, Richards GR, Martens EC, Goodrich-Blair H. 2007. The global regulator Lrp contributes to mutualism, pathogenesis and phenotypic variation in the bacterium Xenorhabdus nematophila. Cell Microbiol 9:1311–1323. doi:10.1111/j.1462-5822.2006.00873.x 17223926

[47] Jin G, Kim IH, Kim Y. 2024. The Lrp transcriptional factor of an entomopathogenic bacterium, Xenorhabdus hominickii, activates non-ribosomal peptide synthetases to suppress insect immunity. Dev Comp Immunol 151:105101. doi:10.1016/j.dci.2023.105101 38000489

[48] Dror B, Wang Z, Brady SF, Jurkevitch E, Cytryn E. 2020. Elucidating the diversity and potential function of nonribosomal peptide and polyketide biosynthetic gene clusters in the root microbiome. mSystems 5:e00866-20. doi:10.1128/mSystems.00866-20 33361322 PMC7762793

[49] Shi YM, Bode HB. 2018. Chemical language and warfare of bacterial natural products in bacteria-nematode-insect interactions. Nat Prod Rep 35:309–335. doi:10.1039/c7np00054e 29359226

[50] Gutiérrez-Chávez C, Benaud N, Ferrari BC. 2021. The ecological roles of microbial lipopeptides: where are we going? Comput Struct Biotechnol J 19:1400–1413. doi:10.1016/j.csbj.2021.02.017 33777336 PMC7960500

[51] Maxwell PW, Chen G, Webster JM, Dunphy GB. 1994. Stability and activities of antibiotics produced during infection of the insect Galleria mellonella by two isolates of Xenorhabdus nematophilus. Appl Environ Microbiol 60:715–721. doi:10.1128/aem.60.2.715-721.1994 16349198 PMC201371

[52] Drace K, Darby C. 2008. The hmsHFRS operon of Xenorhabdus nematophila is required for biofilm attachment to Caenorhabditis elegans. Appl Environ Microbiol 74:4509–4515. doi:10.1128/AEM.00336-08 18515487 PMC2493154

[53] Obeng N, Czerwinski A, Schütz D, Michels J, Leipert J, Bansept F, García García MJ, Schultheiß T, Kemlein M, Fuß J, Tholey A, Traulsen A, Sondermann H, Schulenburg H. 2023. Bacterial c-di-GMP has a key role in establishing host-microbe symbiosis. Nat Microbiol 8:1809–1819. doi:10.1038/s41564-023-01468-x 37653009 PMC10522488

[54] Kissoyan KAB, Drechsler M, Stange EL, Zimmermann J, Kaleta C, Bode HB, Dierking K. 2019. Natural C. elegans microbiota protects against infection via production of a cyclic lipopeptide of the viscosin group. Curr Biol 29:1030–1037. doi:10.1016/j.cub.2019.01.050 30827913

[55] Pradel E, Zhang Y, Pujol N, Matsuyama T, Bargmann CI, Ewbank JJ. 2007. Detection and avoidance of a natural product from the pathogenic bacterium Serratia marcescens by Caenorhabditis elegans. Proc Natl Acad Sci USA 104:2295–2300. doi:10.1073/pnas.0610281104 17267603 PMC1892944

[56] Fischbach MA, Walsh CT, Clardy J. 2008. The evolution of gene collectives: how natural selection drives chemical innovation. Proc Natl Acad Sci USA 105:4601–4608. doi:10.1073/pnas.0709132105 18216259 PMC2290807

[57] Baunach M, Chowdhury S, Stallforth P, Dittmann E. 2021. The landscape of recombination events that create nonribosomal peptide diversity. Mol Biol Evol 38:2116–2130. doi:10.1093/molbev/msab015 33480992 PMC8097286

[58] Booysen E, Rautenbach M, Stander MA, Dicks LMT. 2021. Profiling the production of antimicrobial secondary metabolites by Xenorhabdus khoisanae J194 under different culturing conditions. Front Chem 9:626653. doi:10.3389/fchem.2021.626653 33859975 PMC8042232

[59] Rosa JS, Cabral C, Simões N. 2002. Differences between the pathogenic processes induced by Steinernema and Heterorhabditis (Nemata: Rhabditida) in Pseudaletia unipuncta (Insecta: Lepidoptera). J Invertebr Pathol 80:46–54. doi:10.1016/s0022-2011(02)00038-1 12234542

[60] Ogier JC, Pagès S, Bisch G, Chiapello H, Médigue C, Rouy Z, Teyssier C, Vincent S, Tailliez P, Givaudan A, Gaudriault S. 2014. Attenuated virulence and genomic reductive evolution in the entomopathogenic bacterial symbiont species, Xenorhabdus poinarii. Genome Biol Evol 6:1495–1513. doi:10.1093/gbe/evu119 24904010 PMC4079199

[61] McMullen JG 2nd, Peterson BF, Forst S, Blair HG, Stock SP. 2017. Fitness costs of symbiont switching using entomopathogenic nematodes as a model. BMC Evol Biol 17:100. doi:10.1186/s12862-017-0939-6 28412935 PMC5392933

[62] Heungens K, Cowles CE, Goodrich-Blair H. 2002. Identification of Xenorhabdus nematophila genes required for mutualistic colonization of Steinernema carpocapsae nematodes. Mol Microbiol 45:1337–1353. doi:10.1046/j.1365-2958.2002.03100.x 12207701

[63] Cowles CE, Goodrich-Blair H. 2008. The Xenorhabdus nematophila nilABC genes confer the ability of Xenorhabdus spp. to colonize Steinernema carpocapsae nematodes. J Bacteriol 190:4121–4128. doi:10.1128/JB.00123-08 18390667 PMC2446770

[64] Bhasin A, Chaston JM, Goodrich-Blair H. 2012. Mutational analyses reveal overall topology and functional regions of NilB, a bacterial outer membrane protein required for host association in a model of animal-microbe mutualism. J Bacteriol 194:1763–1776. doi:10.1128/JB.06711-11 22287518 PMC3302474

[65] Grossman AS, Escobar CA, Mans EJ, Mucci NC, Mauer TJ, Jones KA, Moore CC, Abraham PE, Hettich RL, Schneider L, Campagna SR, Forest KT, Goodrich-Blair H. 2022. A surface exposed, two-domain lipoprotein cargo of a type xi secretion system promotes colonization of host intestinal epithelia expressing glycans. Front Microbiol 13:800366. doi:10.3389/fmicb.2022.800366 35572647 PMC9100927

[66] Ausubel FM, Brent R, Kingston RE, Moore DD, Seidman JG, Smith JA, Struhl K. 1988. Current Protocols in Molecular Biology. John Wiley & Sons, Inc, Media, PA.

[67] Mouammine A, Pages S, Lanois A, Gaudriault S, Jubelin G, Bonabaud M, Cruveiller S, Dubois E, Roche D, Legrand L, Brillard J, Givaudan A. 2017. An antimicrobial peptide-resistant minor subpopulation of Photorhabdus luminescens is responsible for virulence. Sci Rep 7:43670. doi:10.1038/srep43670 28252016 PMC5333078

[68] Jubelin G, Lanois A, Severac D, Rialle S, Longin C, Gaudriault S, Givaudan A. 2013. FliZ is a global regulatory protein affecting the expression of flagellar and virulence genes in individual Xenorhabdus nematophila bacterial cells. PLoS Genet 9:e1003915. doi:10.1371/journal.pgen.1003915 24204316 PMC3814329

[69] Vigneux F, Zumbihl R, Jubelin G, Ribeiro C, Poncet J, Baghdiguian S, Givaudan A, Brehélin M. 2007. The xaxAB genes encoding a new apoptotic toxin from the insect pathogen Xenorhabdus nematophila are present in plant and human pathogens. J Biol Chem 282:9571–9580. doi:10.1074/jbc.M604301200 17229739

[70] Givaudan A, Baghdiguian S, Lanois A, Boemare N. 1995. Swarming and swimming changes concomitant with phase variation in Xenorhabdus nematophilus. Appl Environ Microbiol 61:1408–1413. doi:10.1128/aem.61.4.1408-1413.1995 16534993 PMC1388411

[71] Toguchi A, Siano M, Burkart M, Harshey RM. 2000. Genetics of swarming motility in Salmonella enterica serovar typhimurium: critical role for lipopolysaccharide. J Bacteriol 182:6308–6321. doi:10.1128/JB.182.22.6308-6321.2000 11053374 PMC94776

[72] Pothula R, Lee MW, Patricia Stock S. 2023. Type 6 secretion system components hcp and vgrG support mutualistic partnership between Xenorhabdus bovienii symbiont and Steinernema jollieti host. J Invertebr Pathol 198:107925. doi:10.1016/j.jip.2023.107925 37087093

[73] Clinical and Laboratory Standards Institute. 2015. Methods for dilution antimicrobial susceptibility tests for bacteria that grow aerobically, 10th ed. Vol. M07-A10. Wayne, PA Clinical and Laboratory Standards Institute

[74] Duvic B, Jouan V, Essa N, Girard PA, Pagès S, Abi Khattar Z, Volkoff NA, Givaudan A, Destoumieux-Garzon D, Escoubas JM. 2012. Cecropins as a marker of Spodoptera frugiperda immunosuppression during entomopathogenic bacterial challenge. J Insect Physiol 58:881–888. doi:10.1016/j.jinsphys.2012.04.001 22487443

[75] Givaudan A, Lanois A. 2000. flhDC, the flagellar master operon of Xenorhabdus nematophilus: requirement for motility, lipolysis, extracellular hemolysis, and full virulence in insects. J Bacteriol 182:107–115. doi:10.1128/JB.182.1.107-115.2000 10613869 PMC94246

[76] Jubelin G, Pagès S, Lanois A, Boyer MH, Gaudriault S, Ferdy JB, Givaudan A. 2011. Studies of the dynamic expression of the Xenorhabdus FliAZ regulon reveal atypical iron-dependent regulation of the flagellin and haemolysin genes during insect infection. Environ Microbiol 13:986–1000. doi:10.1111/j.1462-2920.2011.02427.x 21332625

[77] Huot L, Bigourdan A, Pagès S, Ogier JC, Girard PA, Nègre N, Duvic B. 2016. Partner-specific induction of Spodoptera frugiperda immune genes in response to the entomopathogenic nematobacterial complex Steinernema carpocapsae–Xenorhabdus nematophila. Dev Comp Immunol 60:112–123. doi:10.1016/j.dci.2016.02.017 32184079

[78] White GF. 1927. A method for obtaining infective nematode larvae from cultures. Science 66:302–303. doi:10.1126/science.66.1709.302-a 17749713

