# Supplementary material for: Pleiotropic role of PAX cyclolipopeptides in the Xenorhabdus bacterium mutualistically associated with entomopathogenic nematodes
Source: Appl Environ Microbiol. 2025 Sep 9;91(10):e00760-25. doi: 10.1128/aem.00760-25 (PMC12542672; doi:10.1128/aem.00760-25)
Supplement: Supplemental material — Figures S1 to S6 and Tables S1 to S5. [file aem.00760-25-s0001.pdf]

Supplementary materials

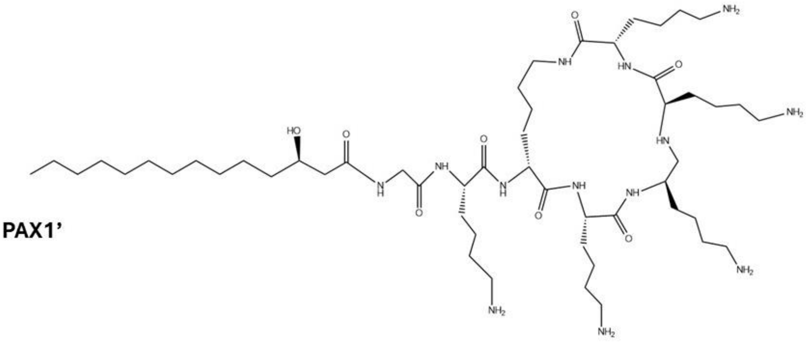

PAX1’

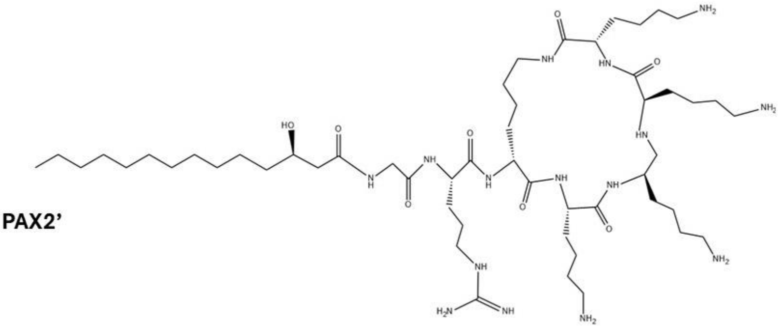

PAX2’

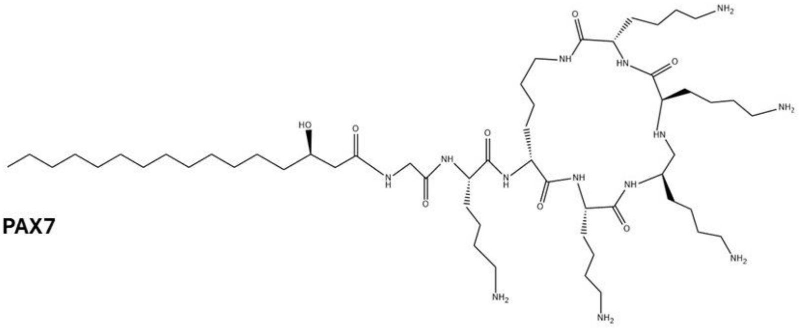

PAX7

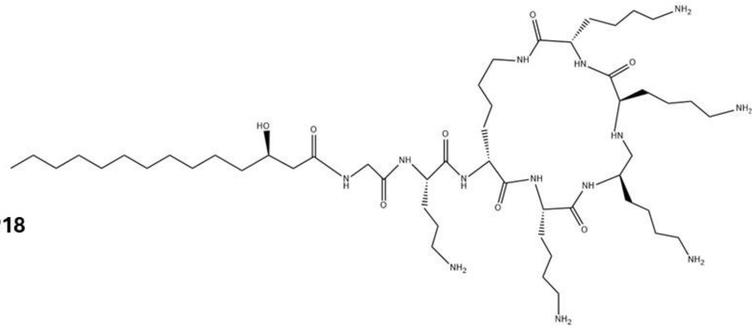

DP18

Figure S1: Structure of the synthetic PAX peptides and derivatives used in this study.
PAX1′: R-3-Hydroxytetradecanoic acid-Gly-Lys-[DLys-Lys-DLys-DLys-Lys],
PAX2′: R-3-Hydroxytetradecanoic acid-Gly-Arg-[DLys-Lys-DLys-DLys-Lys],
PAX7: R-3-Hydroxyhexadecanoic acid-Gly-Lys-[DLys-Lys-DLys-DLys-Lys],
DP18: R-3-Hydroxytetradecanoic acid-Gly-Orn-[DLys-Lys-DLys-DLys-Lys].

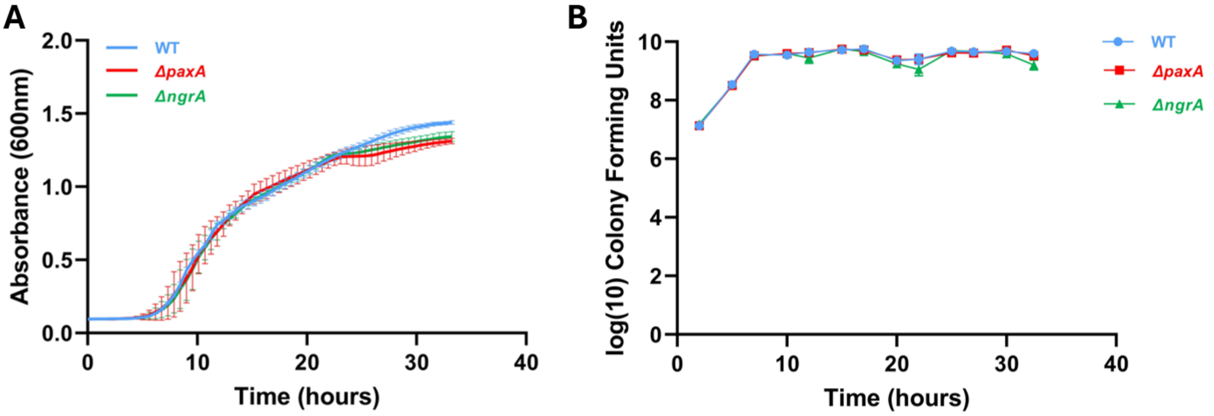

Figure S2: Bacterial growth of *X. nematophila* WT, Δ*paxA* and Δ*ngrA in vitro*. A) A 96-well plate was inoculated with 180 µL of LB and 20 µL of preculture normalized to OD_{600nm}=1 and diluted 1:50. The plate was cultured in a TECAN Infinite® 200 plate reader at 28°C with agitation for 35 h to generate the growth curve. Generation times WT: G=3.07 h, Δ*paxA*: G=2.41h, Δ*ngrA*: G=2.38h. No significant difference in exponential-phase growth or on the whole curve was observed between WT and the Δ*paxA* (p=0.22399) and Δ*ngrA* (p=0.31256) mutants (Wilcoxon-Mann-Whitney test, n=3). **B)** 3 x 300 mL Erlenmeyer flasks were inoculated with 100 mL LB and overnight cultures normalized to OD_{600nm}=1. At each time point, 1 mL was collected to measure OD_{600nm} and plated on GNO medium to assess CFU. The log(10) of CFU values has been calculated. No significant difference in CFU was observed between WT and the Δ*paxA* (p=0.744) and Δ*ngrA* (p=0.174) mutants (Wilcoxon-Mann-Whitney test, n=3).

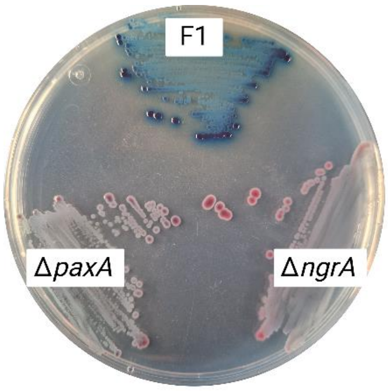

Figure S3: Phenotypes of *X. nematophila* WT, Δ*paxA* and Δ*ngrA* colonies on NBTA medium. Blue colonies display reduction of triphenyl 2.3.5 tetrazolium (TTC) to formazan (red formazan staining masked by blue adsorption) and adsorption of bromothymol blue (BBT). Red colonies display a reduction of TTC to formazan but no BBT adsorption.

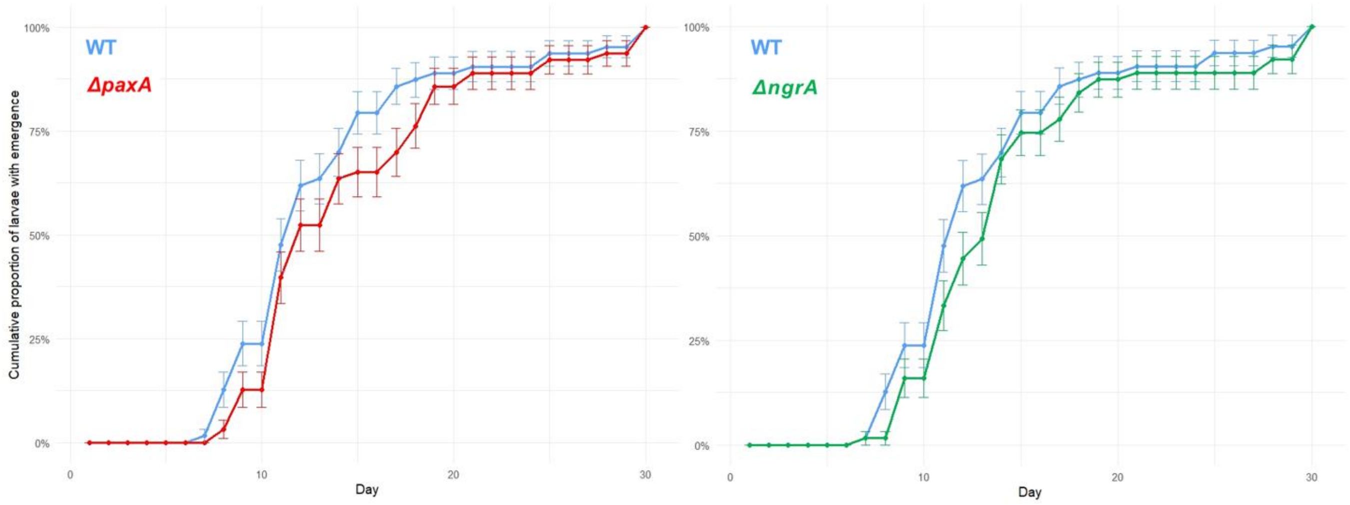

Figure S4: Emergence curves of aposymbiotic nematodes *S. carpocapsae* SK27 reassociated with *X. nematophila* WT, Δ*paxA* or Δ*ngrA* in *G. mellonella* larvae in the 2nd generation. Emerging IJs were monitored from 6 to 30 days post-infestation. Cox model analysis, n=60, $p>0.05$.

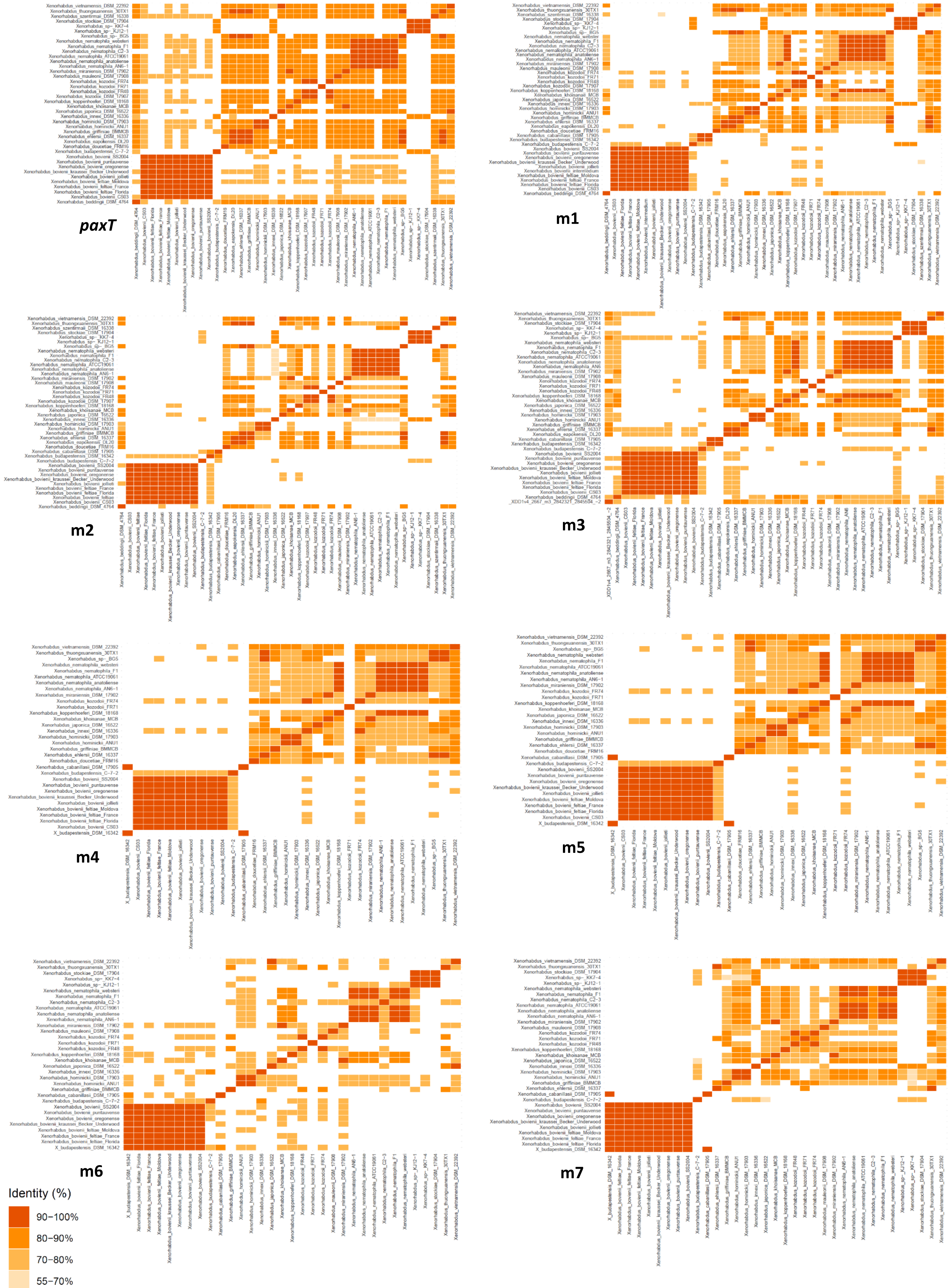
paxT
m1
m2
m3
m4
m5
m6
m7
Identity (%)
90–100%
80–90%
70–80%
55–70%

Figure S5: *paxTABC* gene distance matrix in the *Xenorhabdus* genus. The *paxTABC* sequences of 46 strains belonging to 24 *Xenorhabdus* species were extracted from the MicroScope MaGe database. A blastn analysis using the NCBI Blast+ tool was performed on the Linux platform with a custom script to compare the gene and module sequences of all pairwise strain combinations using default settings. NRPS genes were blasted by module sequences: *paxA*: m1 ; *paxB*: m2, m3, m4 ; *paxC*: m5, m6, m7. Orange square gradient represents percentage identity between each gene and module sequence.

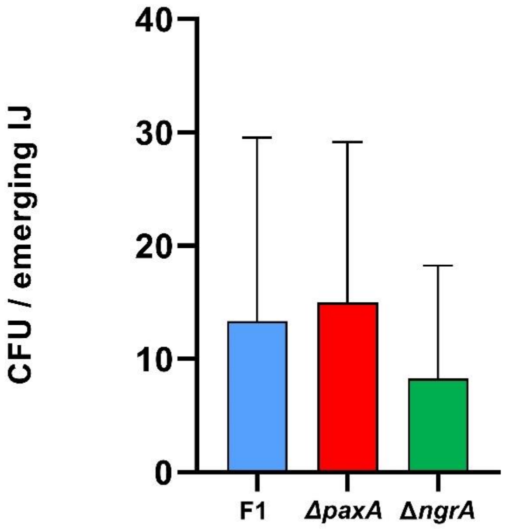

Figure S6: CFU/emerging IJ of aposymbiotic nematodes *S. carpocapsae* SK27 reassociated with *X. nematophila* WT, Δ*paxA* or Δ*ngrA* in *G. mellonella* larvae in the 2nd generation. Emerging IJs were collected and counted at 15- and 30- days post-infestation. Direct counts of average CFU/nematode were determined via a grinding assay wherein collected nematodes were equalized by density, serially diluted, and plated on NBTA plates. Wilcoxon-Mann-Whitney tests, n=60, p>0.05.

Table S1: m/z values of PAX peptides identified by MALDI-TOF-MS in extracts from cultures of *X. nematophila* F1.

PAX peptide name	m/z detected (Da)	m/z literature (Da) (1)
PAX1’	1052.772	1052.793
PAX3’	1066.787	1066.809
PAX6	1078.766	1078.784
PAX2’/PAX7	1080.783	1080.799 / 1080.823
PAX4’	1094.797	1094.815
PAX8	1102.772	1102.807
PAX9	1106.797	1106.815
PAX10	1108.812	1108.831
PAX11	1120.808	1120.831
PAX12	1122.818	1122.845
PAX13	1134.828	1134.846

Table S2: Mass spectra of *in vitro* and *in vivo* PAX peptides production dynamics experiments (Displayed in figure 1).

A) **PAX peptides production *in vitro* (Bacterial cultures in liquid LB medium)**

**20 h A**	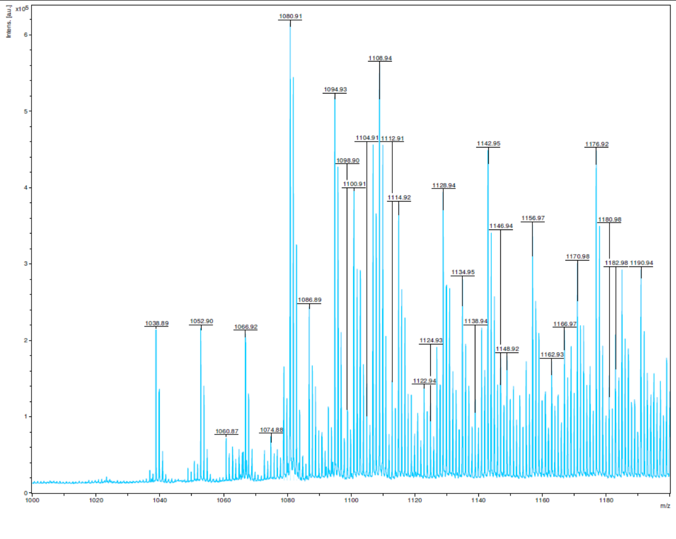 	m/z<br>1038.89<br>1052.90<br>1060.87<br>1066.92<br>1074.88<br>1078.91<br>1080.91<br>1081.92<br>1086.89<br>1088.91<br>1092.92<br>1094.93<br>1098.90<br>1100.91<br>1102.91<br>1104.91<br>1106.93<br>1108.94<br>1112.91<br>1114.92<br>1116.92<br>1120.96<br>1122.94<br>1124.93<br>1126.93<br>1128.94<br>1130.94<br>1134.95<br>1136.94<br>1138.94<br>1140.94<br>1142.95<br>1144.94<br>1146.94<br>1148.92<br>1150.94<br>1152.96<br>1154.96<br>1156.97<br>1158.95<br>1162.93<br>1164.96<br>1166.97<br>1168.97
**20 h B**	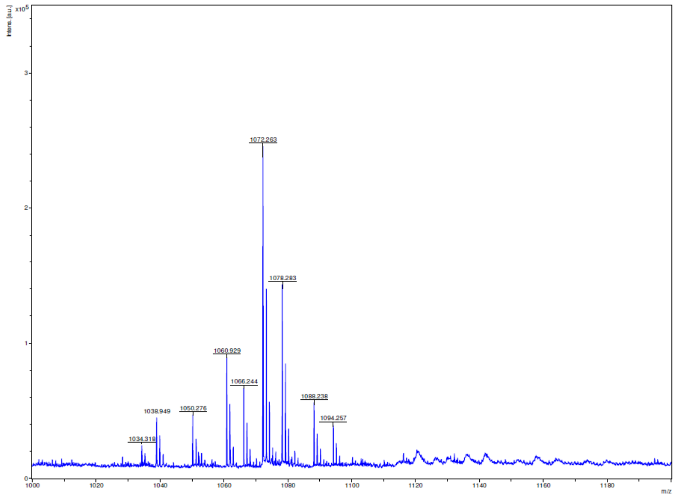 	m/z<br>1034.318<br>1038.949<br>1050.276<br>1052.044<br>1060.929<br>1066.244<br>1072.263<br>1078.283<br>1088.238<br>1094.257
**20 h C**	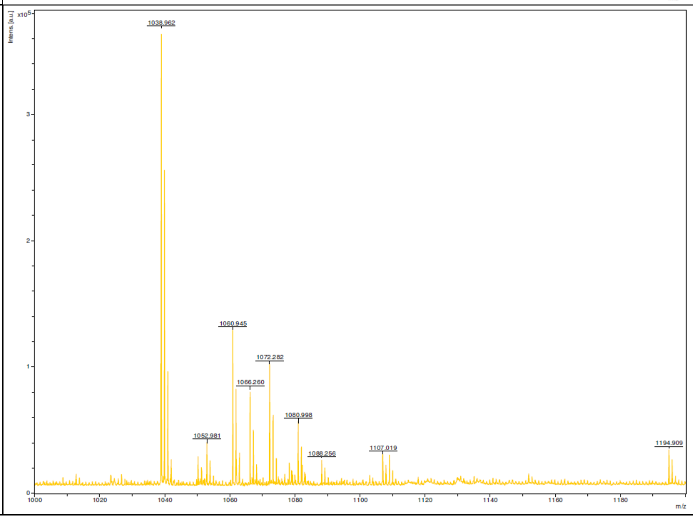 	m/z<br>1038.962<br>1050.292<br>1052.981<br>1060.945<br>1066.260<br>1072.282<br>1078.300<br>1078.993<br>1080.998<br>1082.234<br>1088.256<br>1107.019<br>1109.030<br>1194.909

**48 h A**	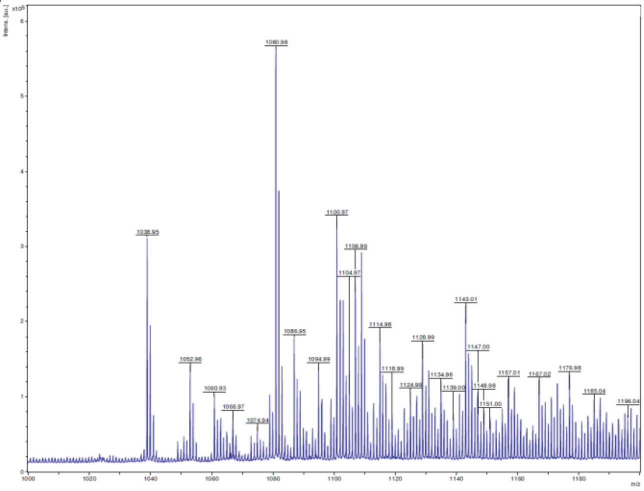	m/z<br>1038.95<br>1052.96<br>1060.93<br>1062.95<br>1066.97<br>1072.93<br>1074.94<br>1078.97<br>1080.98<br>1086.95<br>1092.98<br>1094.99<br>1098.95<br>1100.97<br>1102.97<br>1104.97<br>1106.99<br>1109.01<br>1112.97<br>1114.98<br>1116.98<br>1118.99<br>1122.99<br>1124.99<br>1126.98<br>1128.99<br>1130.99<br>1134.98<br>1139.00<br>1141.00<br>1143.01<br>1144.99<br>1147.00<br>1148.98<br>1151.00<br>1153.01<br>1155.01<br>1157.01<br>1159.00<br>1165.00<br>1167.02<br>1169.02<br>1171.02<br>1173.02
**48 h B**	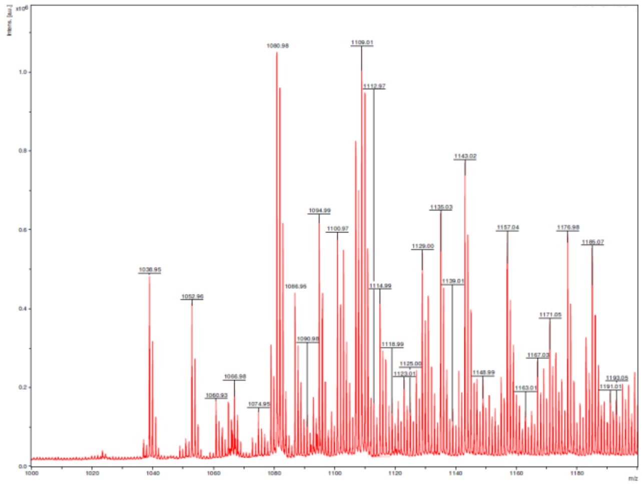	m/z<br>1038.95<br>1052.96<br>1060.93<br>1064.97<br>1066.31<br>1066.98<br>1074.95<br>1078.98<br>1080.98<br>1086.95<br>1088.97<br>1090.98<br>1092.99<br>1094.99<br>1098.96<br>1100.97<br>1102.97<br>1107.00<br>1109.01<br>1111.02<br>1112.97<br>1114.99<br>1116.98<br>1118.99<br>1121.01<br>1123.01<br>1125.00<br>1126.99<br>1129.00<br>1131.00<br>1135.03<br>1139.01<br>1141.01<br>1143.02<br>1145.00<br>1148.99<br>1151.02<br>1155.02<br>1157.04<br>1159.01<br>1163.01<br>1165.02<br>1167.03
**48 h C**	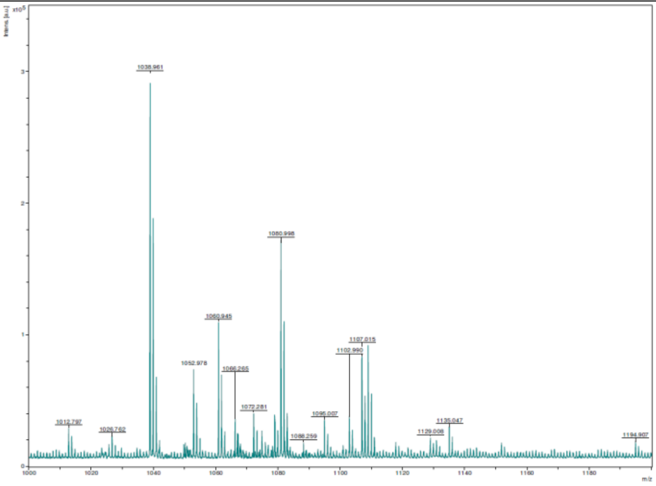	m/z<br>1012.797<br>1026.762<br>1038.961<br>1050.289<br>1052.978<br>1060.945<br>1066.265<br>1066.995<br>1072.281<br>1074.959<br>1078.991<br>1080.998<br>1088.259<br>1095.007<br>1102.990<br>1107.015<br>1109.024<br>1129.008<br>1135.047<br>1194.907

72 h A

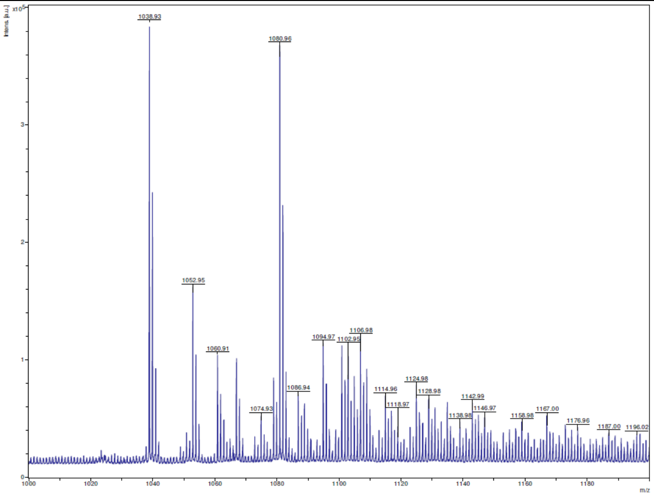

m/z
1038.93
1050.92
1052.95
1060.91
1066.96
1074.93
1078.96
1080.96
1086.94
1088.95
1094.97
1098.94
1100.95
1102.95
1103.95
1106.98
1108.99
1112.95
1114.96
1116.96
1118.97
1122.97
1124.98
1128.98
1130.97
1134.94
1138.98
1140.98
1142.99
1144.97
1146.97
1152.99
1158.98
1167.00
1172.99
1176.96
1187.00
1196.02

72h B

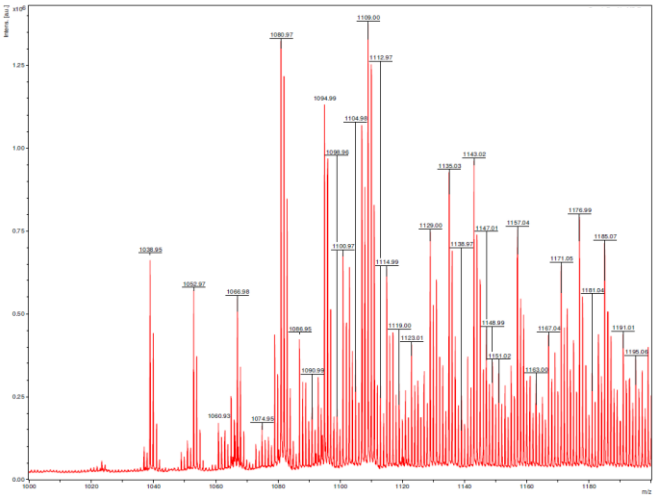

m/z
1038.95
1052.97
1060.93
1064.97
1066.98
1074.95
1078.98
1080.97
1081.99
1086.95
1088.97
1090.99
1092.99
1094.99
1098.96
1100.97
1102.98
1104.98
1107.00
1109.00
1111.02
1112.97
1114.99
1116.99
1119.00
1121.01
1123.01
1125.00
1127.00
1129.00
1131.00
1135.03
1137.02
1138.97
1141.01
1143.02
1145.01
1147.01
1148.99
1151.02
1153.03
1155.03
1157.04

72 h C

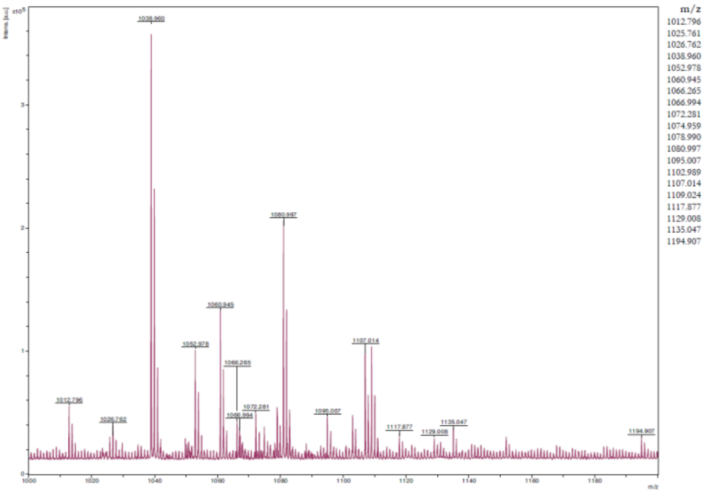

m/z
1012.796
1025.761
1026.762
1038.960
1052.978
1060.945
1066.265
1066.994
1072.281
1074.959
1078.990
1080.997
1095.007
1102.989
1107.014
1109.024
1117.877
1129.008
1135.047
1194.907

**5 d A**	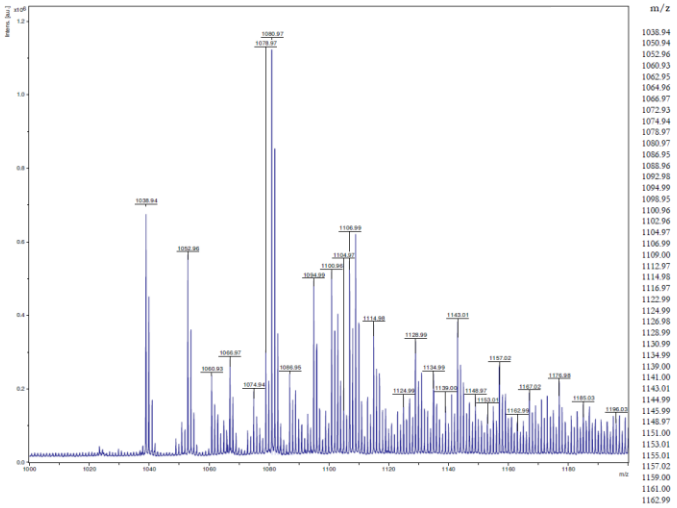m/z<br>1038.94<br>1050.94<br>1052.96<br>1060.93<br>1062.95<br>1064.96<br>1066.97<br>1072.93<br>1074.94<br>1078.97<br>1080.97<br>1086.95<br>1088.96<br>1092.98<br>1094.99<br>1098.95<br>1100.96<br>1102.96<br>1104.97<br>1106.99<br>1109.00<br>1112.97<br>1114.98<br>1116.97<br>1122.99<br>1124.99<br>1126.98<br>1128.99<br>1130.99<br>1134.99<br>1139.00<br>1141.00<br>1143.01<br>1144.99<br>1145.99<br>1148.97<br>1151.00<br>1153.01<br>1155.01<br>1157.02<br>1159.00<br>1161.00<br>1162.99
**5 d B**	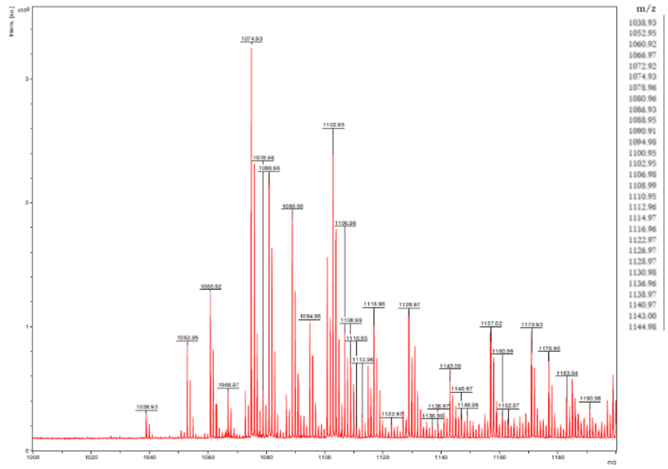m/z<br>1038.93<br>1052.95<br>1060.92<br>1066.97<br>1072.92<br>1074.93<br>1078.96<br>1080.96<br>1086.93<br>1088.95<br>1090.91<br>1094.98<br>1100.95<br>1102.95<br>1106.98<br>1108.99<br>1110.95<br>1112.96<br>1114.97<br>1116.96<br>1122.97<br>1126.97<br>1128.97<br>1130.98<br>1136.96<br>1138.97<br>1140.97<br>1143.00<br>1144.98
**5 d C**	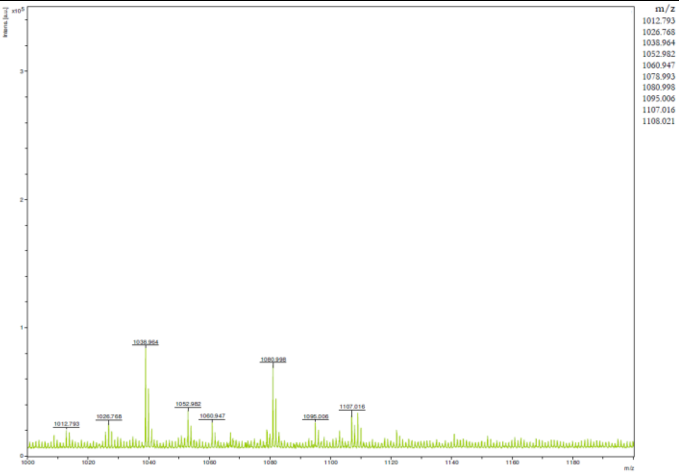m/z<br>1012.793<br>1026.768<br>1038.964<br>1052.982<br>1060.947<br>1078.993<br>1080.998<br>1095.006<br>1107.016<br>1108.021

7 d A

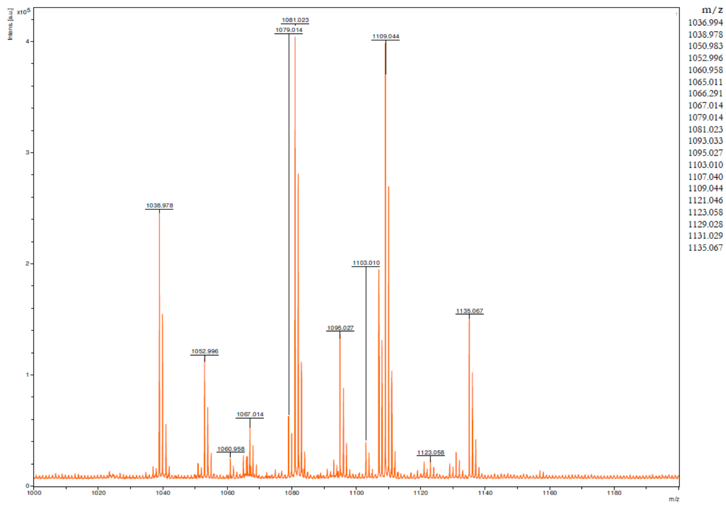

m/z
1036.994
1038.978
1050.983
1052.996
1060.958
1065.011
1066.291
1067.014
1079.014
1081.023
1093.033
1095.027
1103.010
1107.040
1109.044
1121.046
1123.058
1129.028
1131.029
1135.067

7 d B

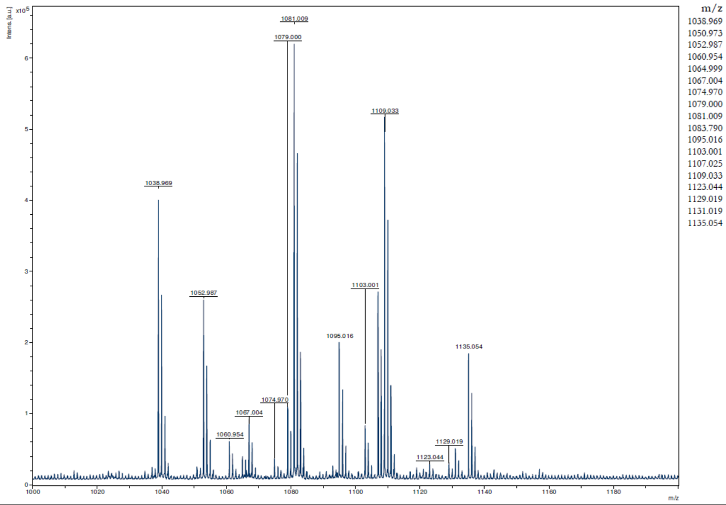

m/z
1038.969
1050.973
1052.987
1060.954
1064.999
1067.004
1074.970
1079.000
1081.009
1083.790
1095.016
1103.001
1107.025
1109.033
1123.044
1129.019
1131.019
1135.054

7 d C

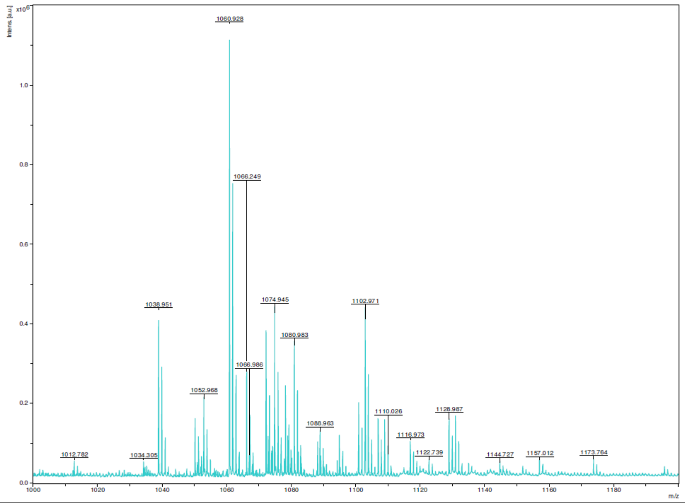

m/z
1012.782
1034.305
1038.951
1050.274
1050.947
1052.968
1060.928
1066.249
1066.986
1072.270
1072.930
1074.945
1078.289
1080.983
1082.223
1088.246
1088.963
1094.263
1094.998
1100.964
1102.971
1110.026
1116.973
1122.739
1128.987
1129.990
1131.999
1144.727
1157.012
1173.764

B) PAX peptides production *in vivo* (Larvae infected with bacteria)

20 h A

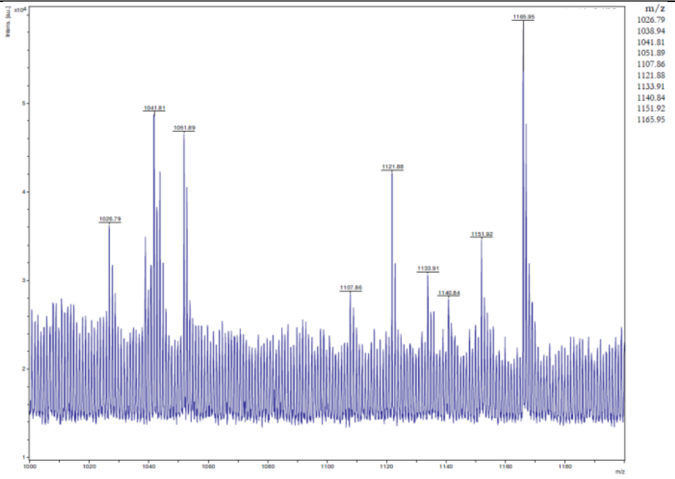

20 h B

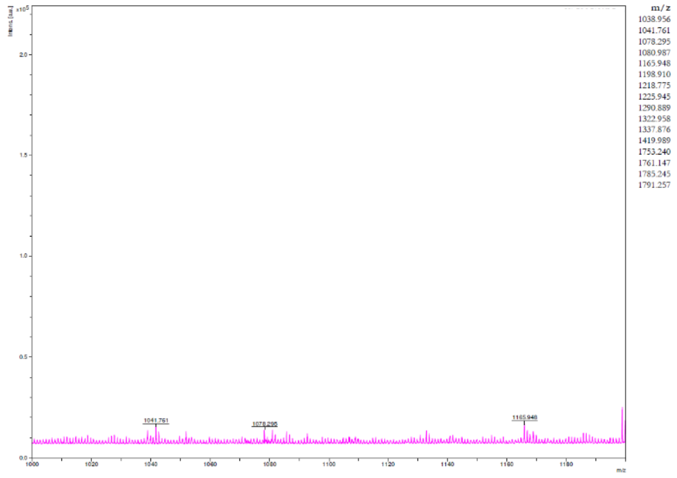

20 h C

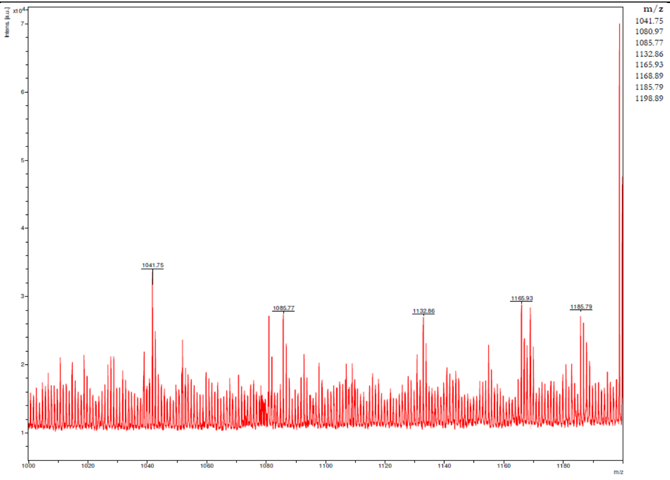

<table>
<tr>
<td>48 h A</td>
<td>
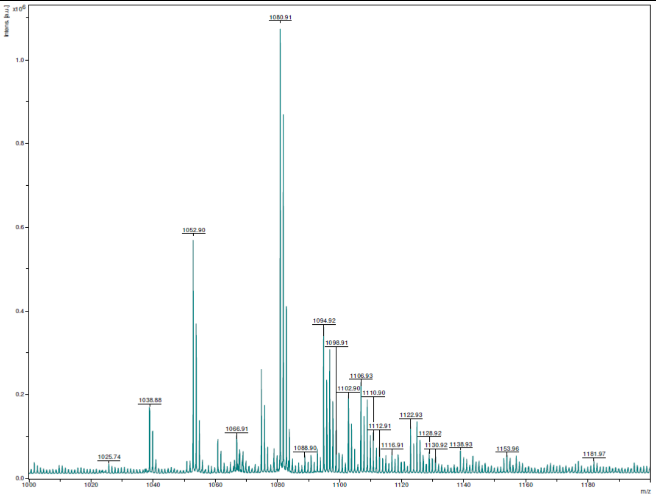

</td>
<td>m/z
1001.77
1025.74
1038.88
1050.88
1052.90
1060.87
1064.89
1066.25
1066.91
1067.70
1068.90
1080.91
1088.90
1090.87
1092.92
1094.92
1096.91
1098.91
1102.90
1106.93
1108.94
1110.90
1112.91
1116.91
1118.90
1122.93
1124.94
1128.92
1130.92
1138.93
1142.94
1151.95
1153.96
1154.82
1156.95
1181.97</td>
</tr>
<tr>
<td>48 h B</td>
<td>
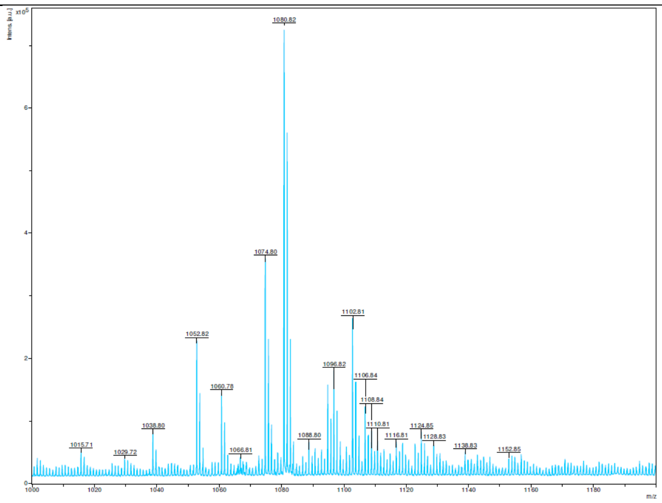

</td>
<td>m/z
1001.69
1015.71
1029.72
1038.80
1052.82
1060.78
1066.81
1074.80
1080.82
1088.80
1090.79
1094.83
1096.82
1100.81
1102.81
1106.84
1108.84
1110.81
1116.81
1118.81
1122.83
1124.85
1128.83
1138.83
1142.83
1152.85</td>
</tr>
<tr>
<td>48 h C</td>
<td>
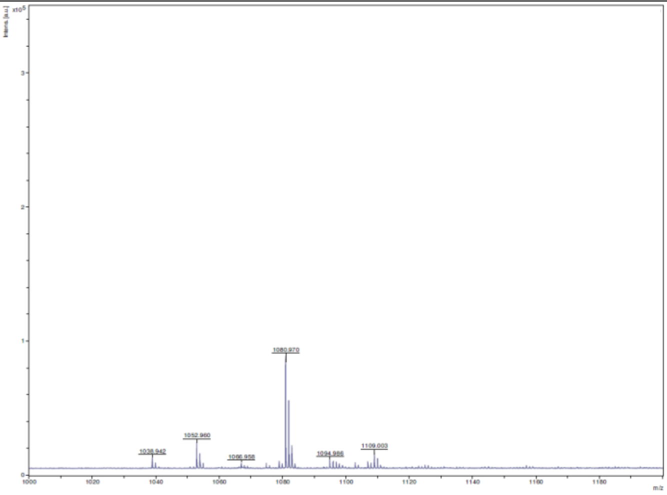

</td>
<td>m/z
1038.942
1052.960
1066.958
1074.943
1078.953
1080.970
1094.986
1096.964
1102.955
1106.986
1109.003</td>
</tr>
</table>

72 h A

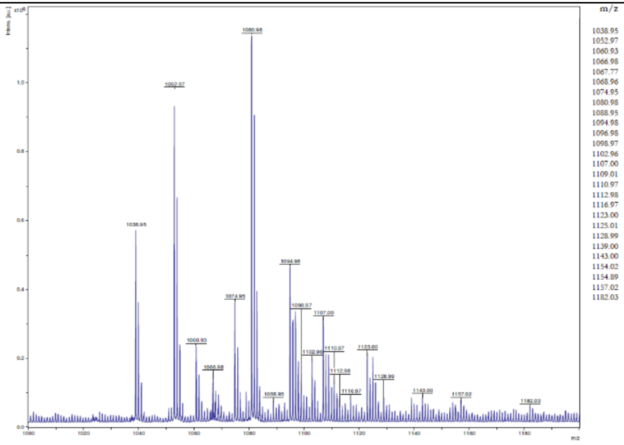

72h B

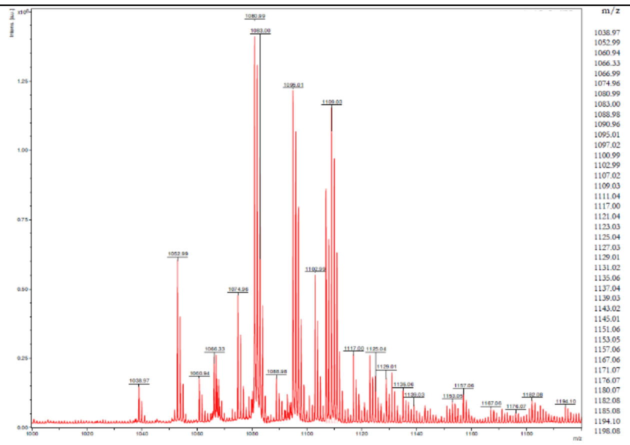

72 h C

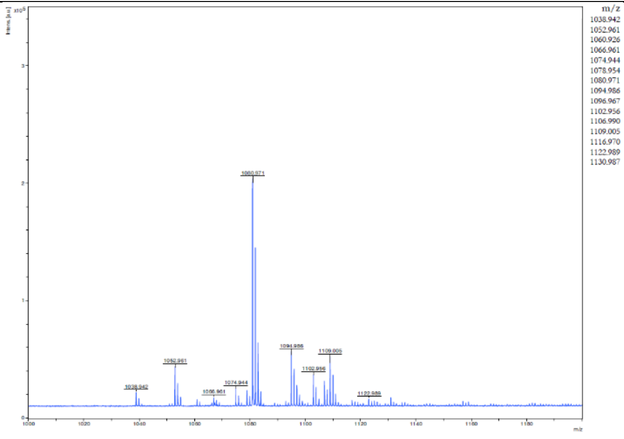

**5 d A**	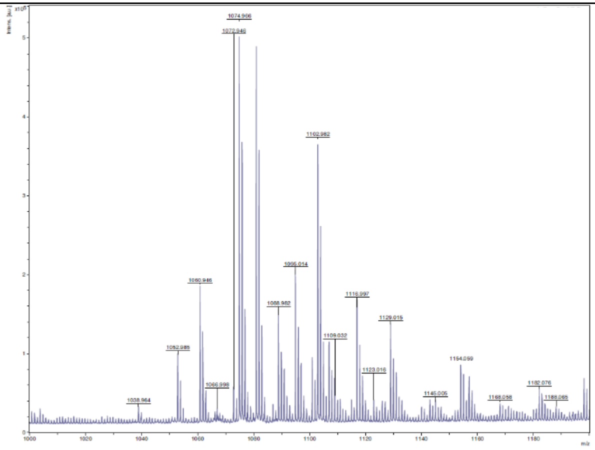<br>m/z<br>1003.815<br>1038.964<br>1052.985<br>1060.946<br>1066.998<br>1072.946<br>1074.966<br>1080.996<br>1086.965<br>1088.982<br>1090.959<br>1095.014<br>1096.996<br>1100.987<br>1102.982<br>1107.018<br>1109.032<br>1111.005<br>1114.998<br>1116.997<br>1118.976<br>1123.016<br>1126.023<br>1129.015<br>1131.015<br>1140.037<br>1143.027<br>1145.005<br>1154.059<br>1157.051<br>1168.058<br>1171.056<br>1182.076<br>1188.065<br>1198.061
**5 d B**	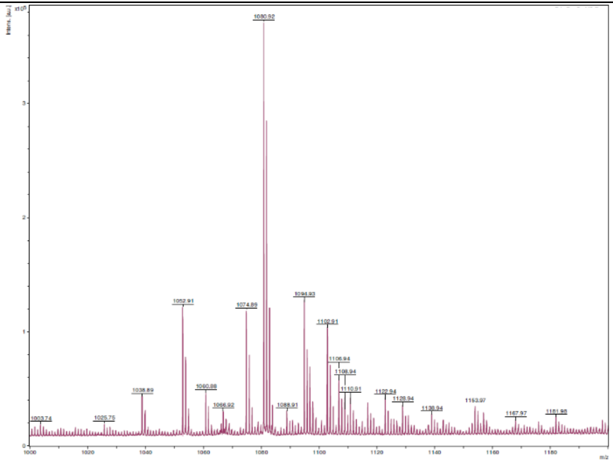<br>m/z<br>1003.74<br>1025.75<br>1038.89<br>1052.91<br>1060.88<br>1066.92<br>1074.89<br>1080.92<br>1088.91<br>1094.93<br>1096.92<br>1100.91<br>1102.91<br>1106.94<br>1108.94<br>1110.91<br>1116.92<br>1122.94<br>1125.94<br>1128.94<br>1138.94<br>1142.94<br>1153.97<br>1156.96<br>1167.97<br>1181.98
**5 d C**	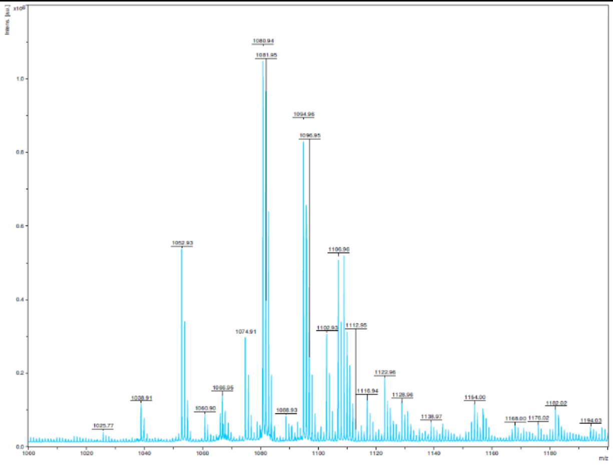<br>m/z<br>1025.77<br>1038.91<br>1052.93<br>1060.90<br>1066.27<br>1066.95<br>1074.91<br>1080.94<br>1081.95<br>1083.72<br>1088.93<br>1091.92<br>1092.95<br>1094.96<br>1096.95<br>1100.94<br>1102.93<br>1106.96<br>1108.97<br>1110.95<br>1112.95<br>1114.95<br>1116.94<br>1118.92<br>1122.96<br>1124.97<br>1126.96<br>1128.96<br>1130.96<br>1134.98<br>1138.97<br>1142.98<br>1151.99<br>1154.00<br>1156.99<br>1168.00<br>1171.00<br>1176.02<br>1180.01<br>1182.02<br>1194.03<br>1198.01

**7 d A**	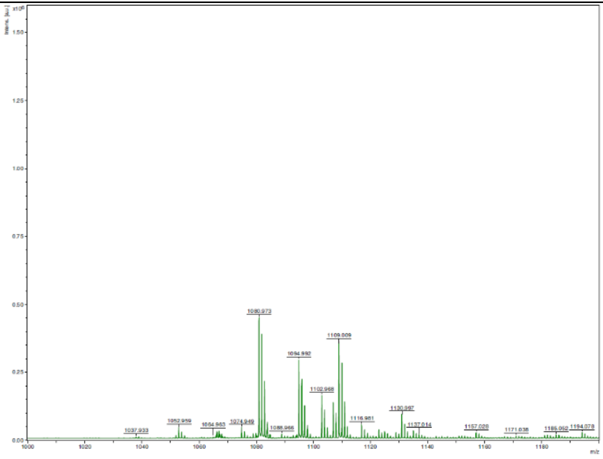<br>m/z<br>1037.933<br>1050.972<br>1051.931<br>1052.959<br>1060.929<br>1064.963<br>1066.314<br>1066.970<br>1074.949<br>1080.973<br>1088.966<br>1091.969<br>1094.992<br>1096.986<br>1102.968<br>1106.998<br>1109.009<br>1110.818<br>1111.015<br>1116.981<br>1123.006<br>1125.010<br>1128.989<br>1130.997<br>1135.027<br>1137.014<br>1138.998<br>1143.010<br>1145.000<br>1150.017<br>1152.025
**7 d B**	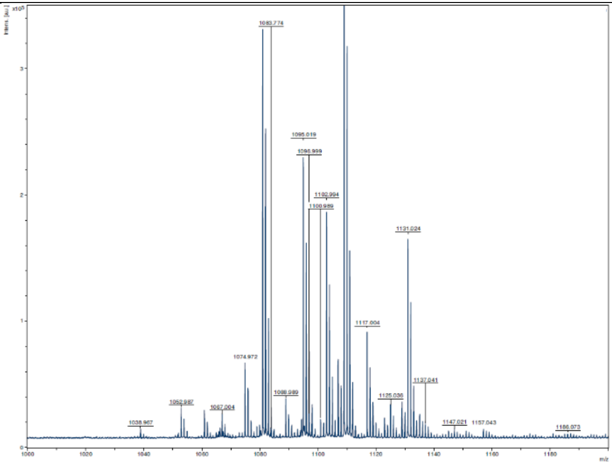<br>m/z<br>1038.967<br>1052.987<br>1060.954<br>1066.349<br>1067.004<br>1072.955<br>1074.972<br>1081.004<br>1083.774<br>1088.989<br>1095.019<br>1096.999<br>1100.989<br>1102.994<br>1109.039<br>1117.004<br>1123.035<br>1125.036<br>1129.008<br>1131.024<br>1135.057<br>1137.041<br>1145.019<br>1147.021<br>1151.057<br>1157.043<br>1186.073
**7 d C**	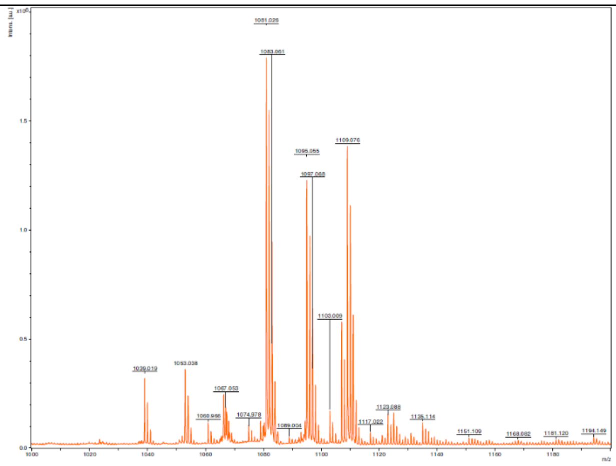<br>m/z<br>1039.019<br>1053.038<br>1060.966<br>1066.364<br>1067.053<br>1074.978<br>1080.399<br>1081.026<br>1083.061<br>1089.004<br>1092.068<br>1095.055<br>1097.068<br>1103.009<br>1107.072<br>1109.076<br>1111.103<br>1117.022<br>1121.084<br>1123.088<br>1125.096<br>1129.044<br>1131.054<br>1135.114<br>1137.098<br>1139.087<br>1143.077<br>1151.109<br>1153.076<br>1157.099<br>1168.082<br>1181.120<br>1194.149

C) PAX peptides production *in vivo* (Larvae infested with nematodes)

**20 h A**	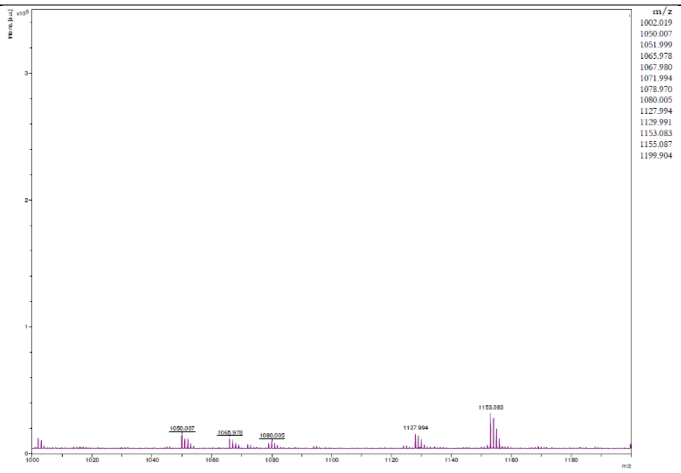
**20 h B**	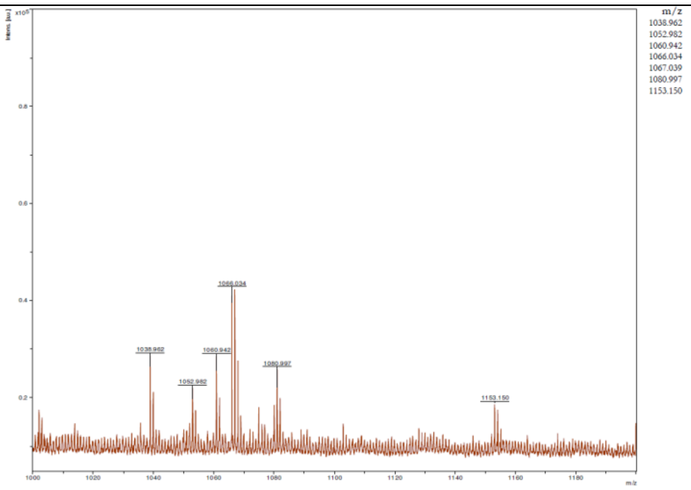
**20 h C**	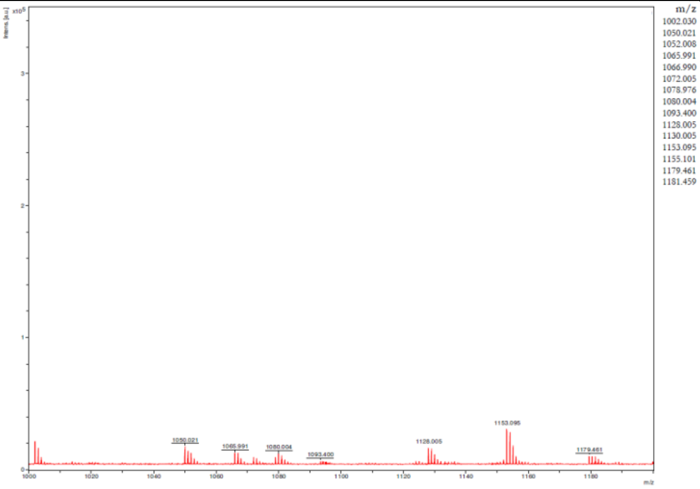

**48 h A**	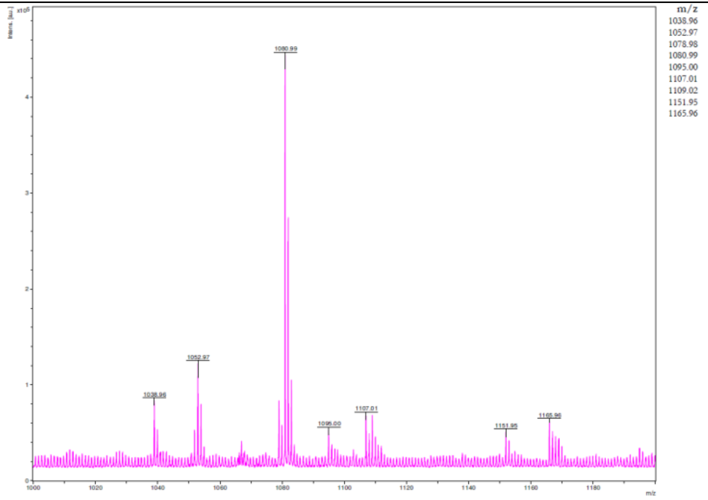
**48 h B**	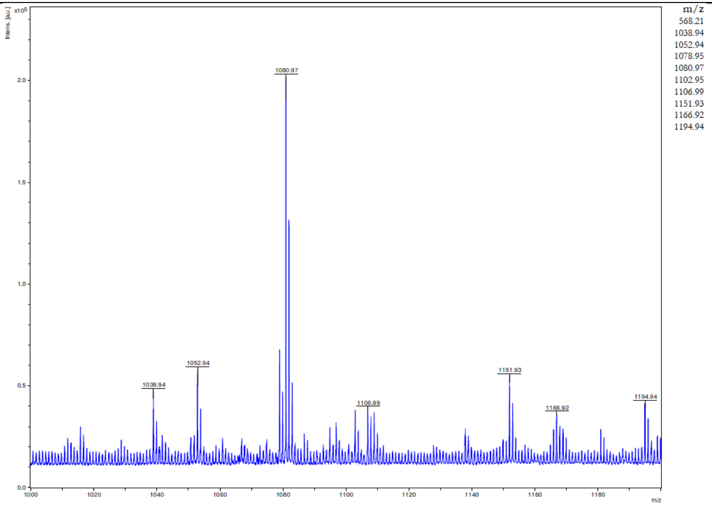
**48 h C**	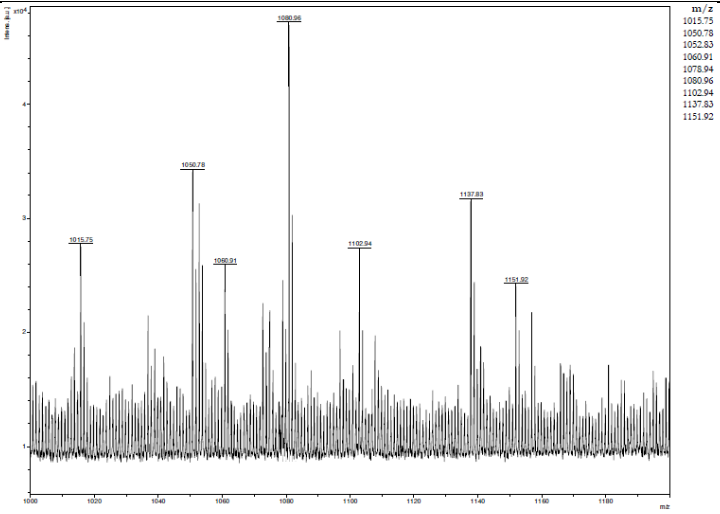

72 h A

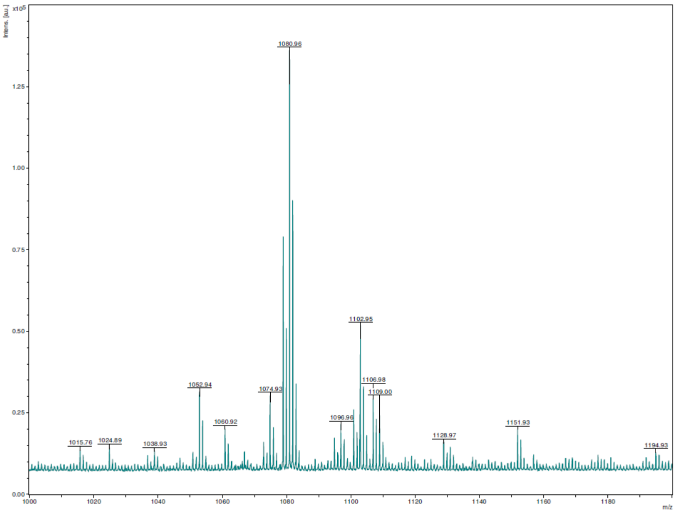

m/z
1015.76
1024.89
1038.93
1052.94
1060.92
1072.92
1074.93
1078.95
1080.96
1094.96
1096.96
1100.94
1102.95
1106.98
1109.00
1128.97
1130.98
1151.93
1194.93

72h B

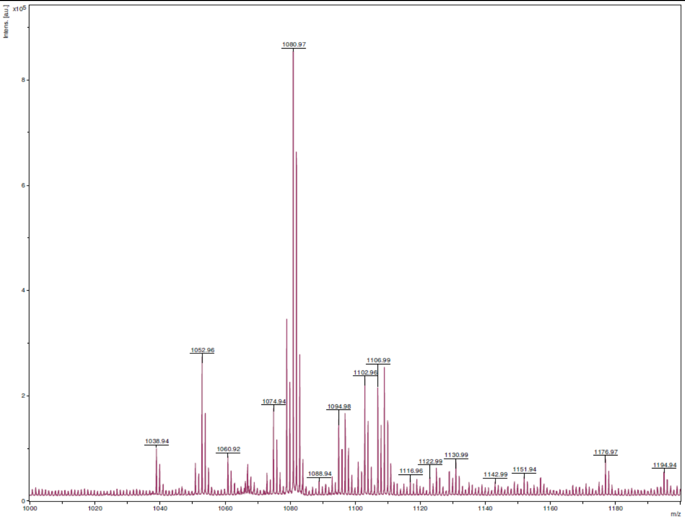

m/z
1038.94
1050.94
1052.96
1060.92
1066.31
1066.96
1072.92
1074.94
1078.96
1080.97
1088.94
1092.97
1094.98
1096.97
1100.95
1102.96
1106.99
1109.00
1116.96
1122.99
1125.00
1128.98
1130.99
1142.99
1148.95
1151.94
1157.00
1174.96
1176.97
1194.94

72 h C

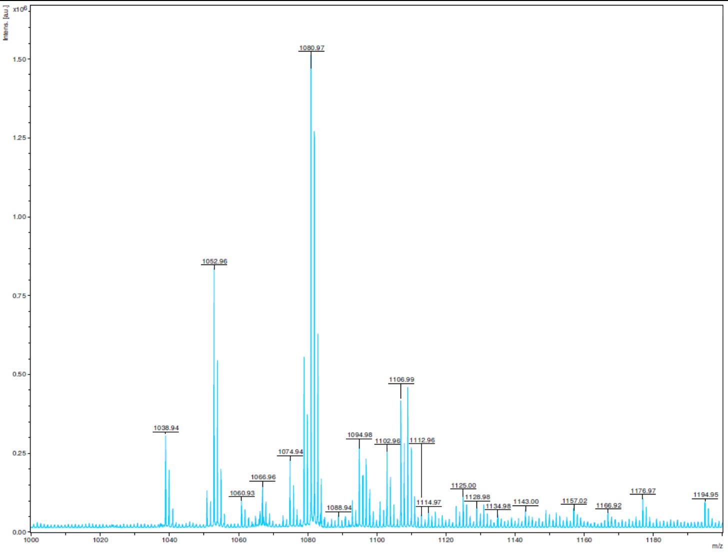

m/z
1038.94
1050.94
1052.96
1060.93
1064.95
1066.30
1066.96
1072.92
1074.94
1078.96
1080.97
1088.94
1092.97
1094.98
1096.97
1100.96
1102.96
1106.99
1109.00
1112.96
1114.97
1116.96
1118.96
1122.99
1125.00
1128.98
1130.99
1134.98
1143.00
1148.96
1151.94
1157.02
1166.92
1176.97
1194.95

5 d A

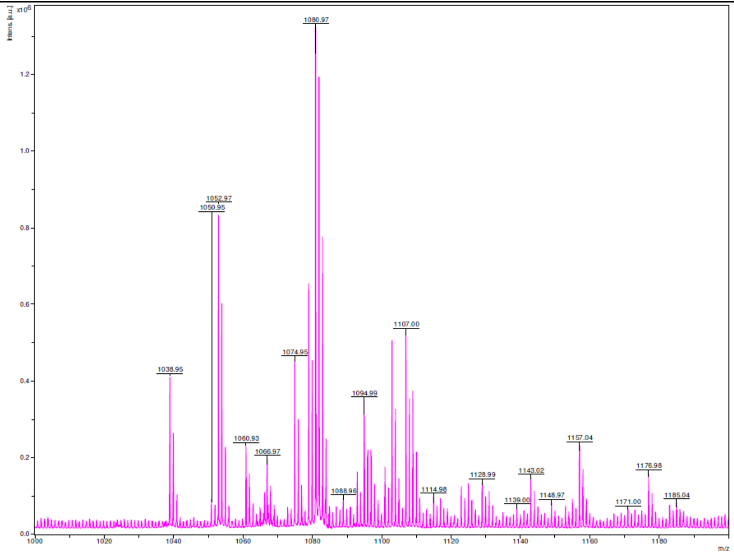

m/z
1038.95
1050.95
1052.97
1060.93
1066.31
1066.97
1072.92
1074.95
1078.97
1080.97
1088.96
1092.98
1094.99
1096.98
1100.97
1102.97
1107.00
1109.01
1114.98
1116.97
1123.00
1125.01
1128.99
1131.00
1139.00
1143.02
1148.97
1153.01
1155.02
1157.04
1171.00
1176.98
1183.04
1185.04

5 d B

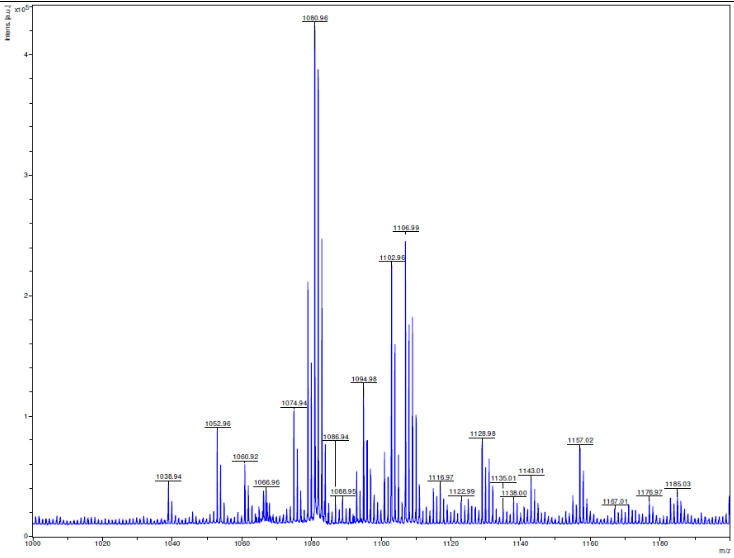

m/z
1038.94
1052.96
1060.92
1064.95
1066.30
1066.96
1072.92
1074.94
1078.96
1080.96
1086.94
1088.95
1092.97
1094.98
1100.95
1102.96
1106.99
1109.00
1114.97
1116.97
1122.99
1125.00
1128.98
1130.99
1135.01
1138.00
1140.99
1143.01
1155.01
1157.02
1167.01
1171.02
1176.97
1183.04
1185.03
1199.90

5 d C

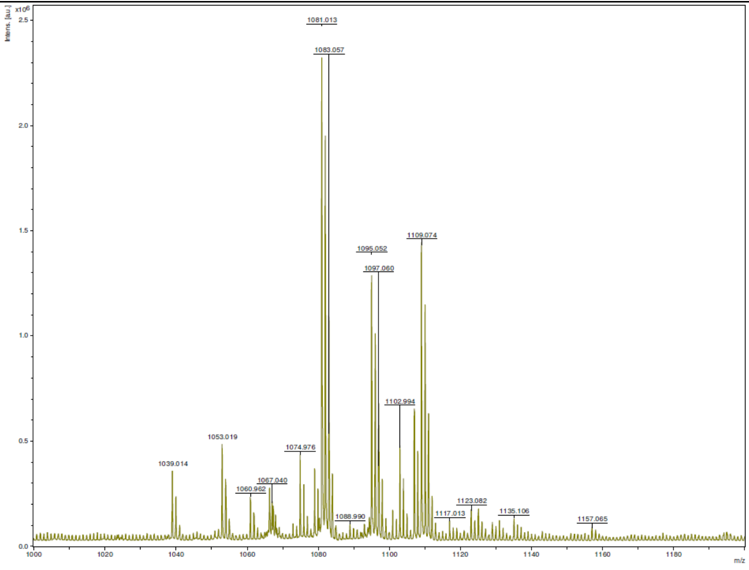

m/z
1039.014
1053.019
1060.962
1066.372
1067.040
1072.956
1074.976
1081.013
1083.057
1088.990
1095.052
1097.060
1100.989
1102.994
1107.065
1109.074
1111.102
1117.013
1123.082
1125.086
1129.026
1131.033
1135.106
1157.065

7 d A

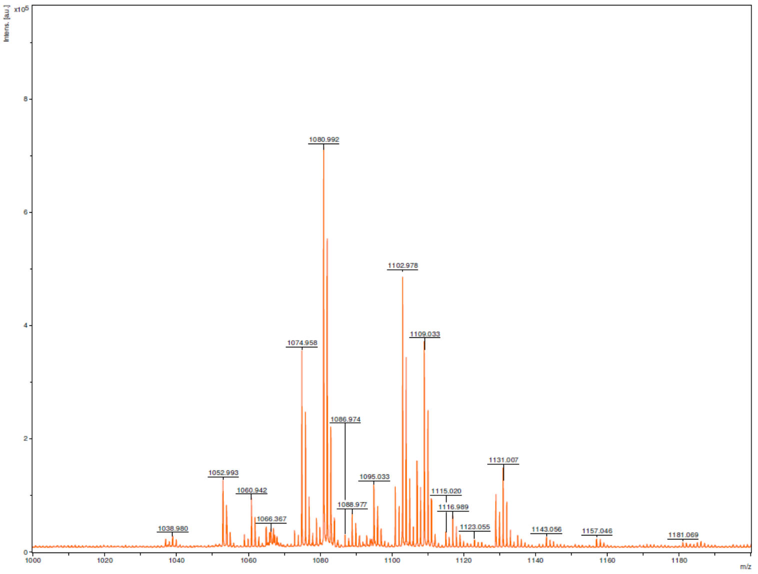

m/z
1037.009
1038.980
1052.993
1058.960
1060.942
1065.000
1066.367
1067.027
1072.942
1074.958
1080.992
1083.816
1086.974
1088.977
1093.031
1095.033
1100.977
1102.978
1109.033
1115.020
1116.989
1118.958
1123.055
1128.993
1131.007
1135.072
1143.056
1157.046
1181.069
1186.090

7 d B

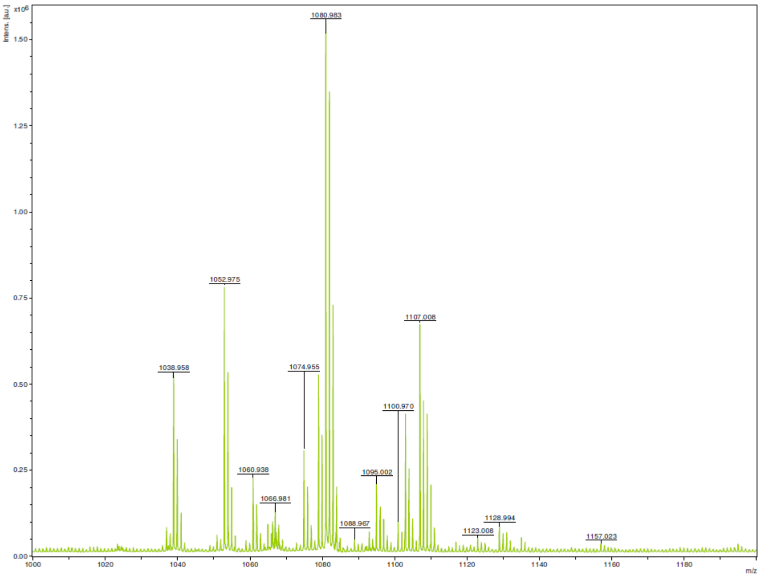

m/z
1036.973
1038.958
1049.962
1052.975
1058.953
1060.938
1064.983
1066.315
1066.981
1074.955
1078.978
1080.983
1088.967
1092.992
1095.002
1096.988
1100.970
1102.973
1107.008
1109.022
1116.980
1123.008
1128.994
1131.004
1135.038
1157.023

7 d C

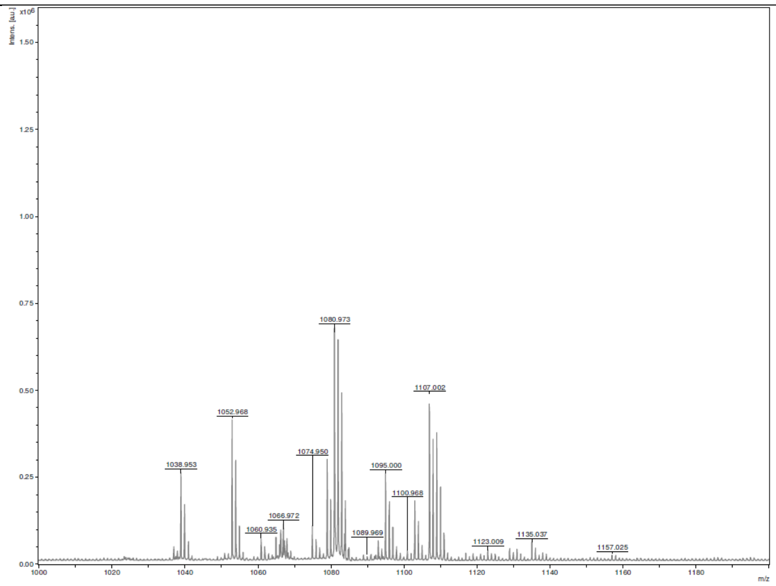

m/z
1036.972
1038.953
1049.960
1052.968
1060.935
1062.973
1064.978
1066.310
1066.972
1074.950
1078.974
1080.973
1081.982
1082.987
1083.777
1089.969
1092.989
1095.000
1096.987
1100.968
1102.971
1107.002
1109.017
1116.980
1118.947
1121.013
1123.009
1125.012
1128.996
1131.001
1135.037
1138.017
1157.025

Table S3: Mass spectra of PAX peptides production in extracts from different strains of *Xenorhabdus* (Displayed in Table 4).

X. bovienii feltiae France	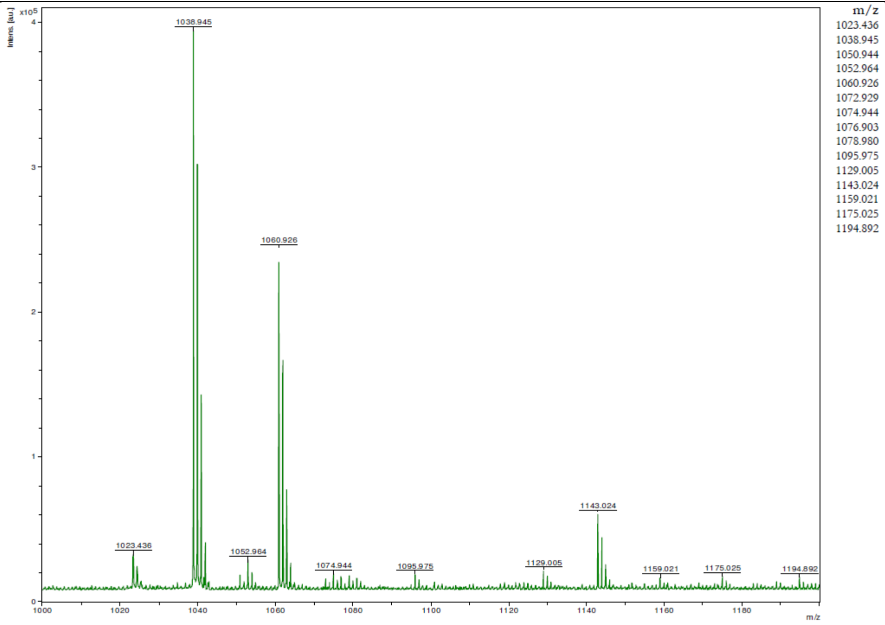
X. budapestensis DSM 16342	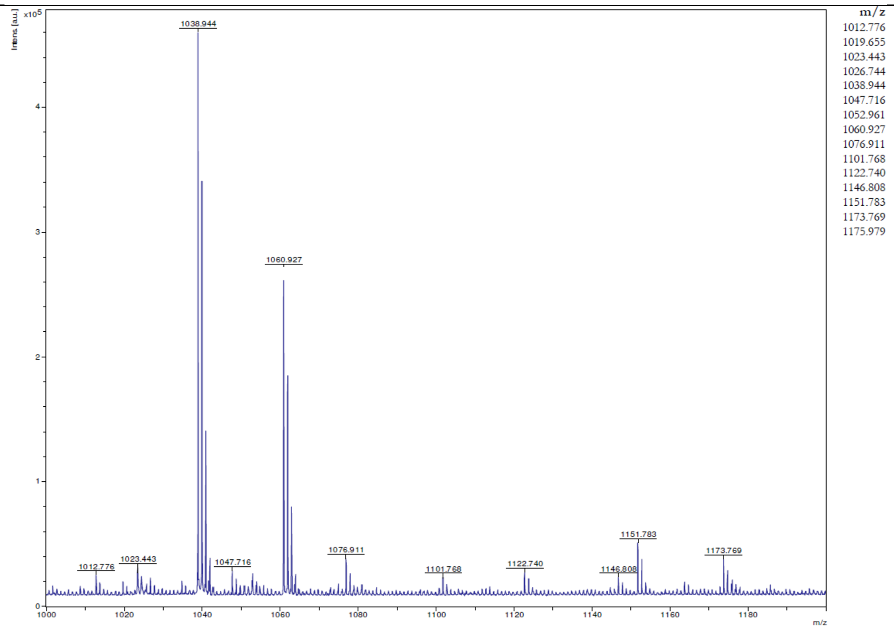

X. cabanillasii<br>DSM 17905	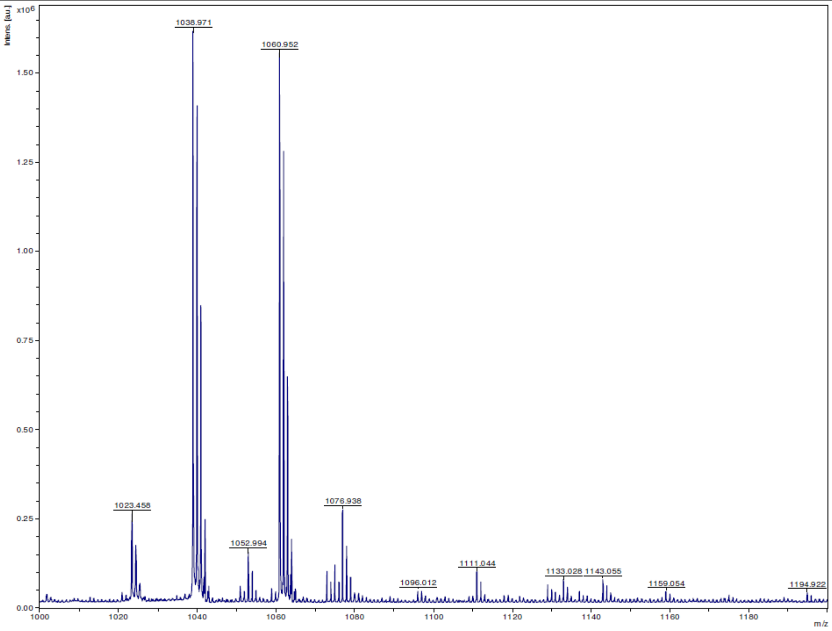<br><br>**m/z**<br>1020.953<br>1023.458<br>1038.971<br>1039.975<br>1041.713<br>1050.976<br>1052.994<br>1058.954<br>1060.952<br>1061.966<br>1072.956<br>1073.963<br>1074.997<br>1076.938<br>1096.012<br>1111.044<br>1129.039<br>1133.028<br>1137.066<br>1143.055<br>1159.054<br>1194.922
X. hominickii<br>DSM 17903	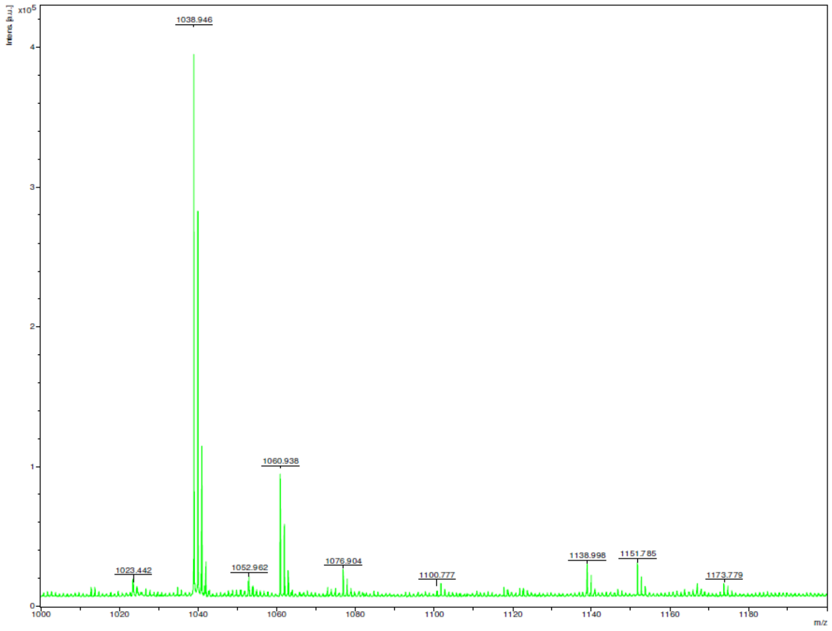<br><br>**m/z**<br>1023.442<br>1038.946<br>1052.795<br>1052.962<br>1060.938<br>1072.945<br>1076.904<br>1100.777<br>1138.998<br>1151.785<br>1167.010<br>1173.779

<table>
<tr>
<td>X. ishibashii
DSM 22670</td>
<td>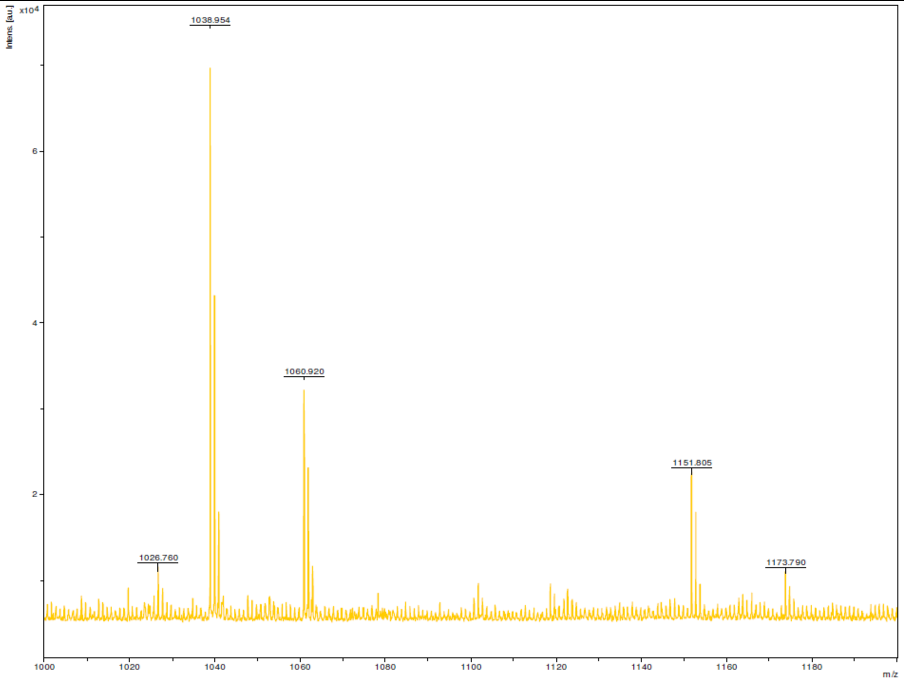
</td>
<td>m/z
1026.760
1038.954
1060.920
1151.805
1173.790</td>
</tr>
<tr>
<td>X. mauleonii
DSM 17908</td>
<td>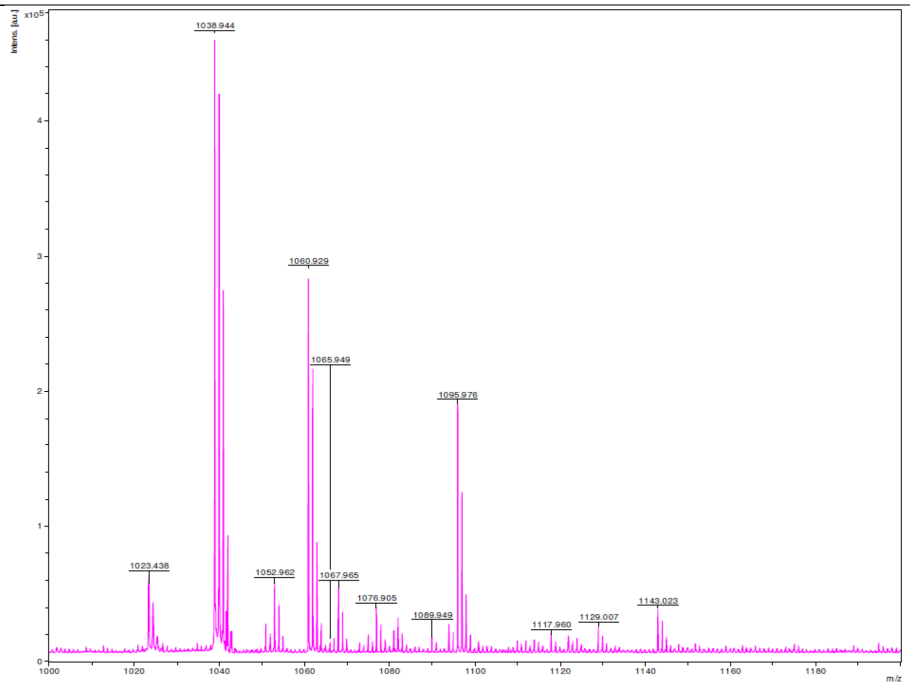
</td>
<td>m/z
1023.438
1026.746
1038.944
1039.947
1050.947
1052.962
1060.929
1065.949
1067.965
1074.944
1076.905
1080.966
1081.961
1089.949
1093.965
1095.976
1113.986
1117.960
1121.849
1121.997
1124.005
1129.007
1143.023</td>
</tr>
</table>

X. nematophila F1 WT

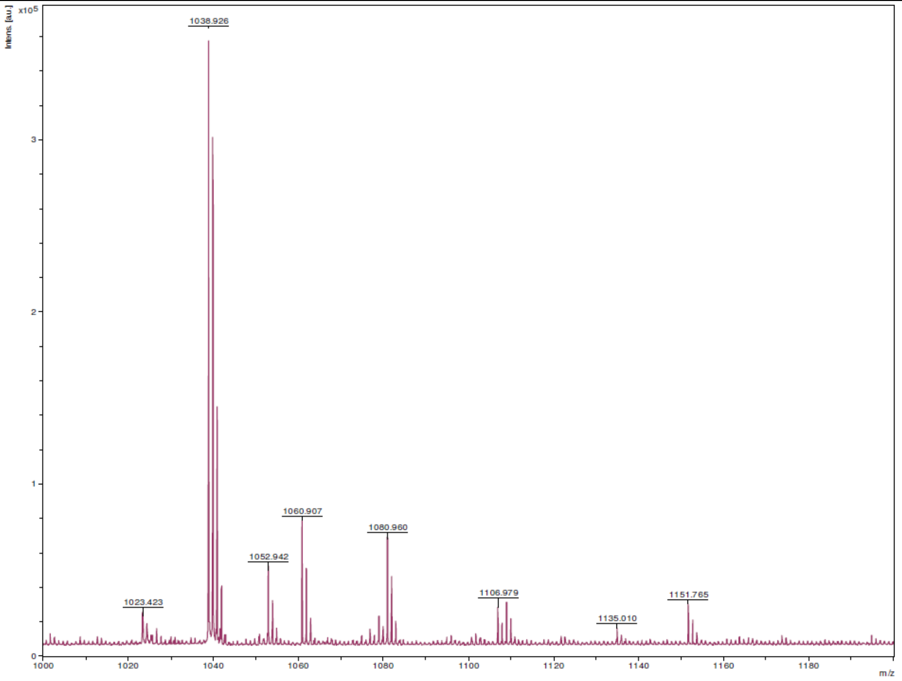

m/z
1023.423
1026.722
1038.926
1052.942
1060.907
1076.885
1078.952
1080.960
1106.979
1108.989
1135.010
1151.765

X. nematophila F1 ΔngrA

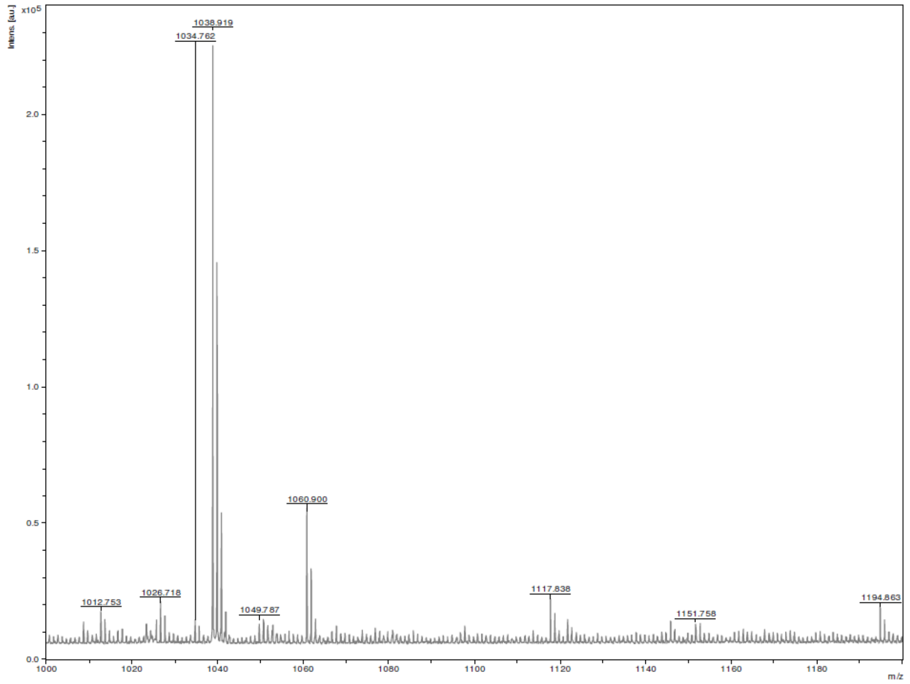

m/z
1008.707
1012.753
1026.718
1034.762
1038.919
1049.787
1060.900
1117.838
1121.815
1151.758
1194.863

X. nematophila F1 ΔpaxA	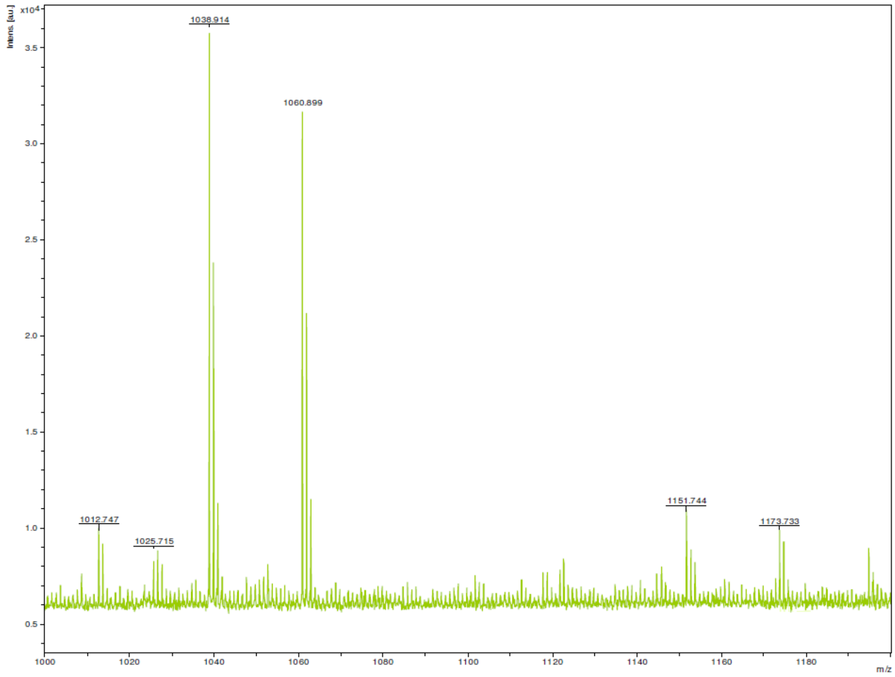	m/z<br>1012.747<br>1025.715<br>1038.914<br>1060.899<br>1151.744<br>1173.733
X. nematophila F1 variant 2	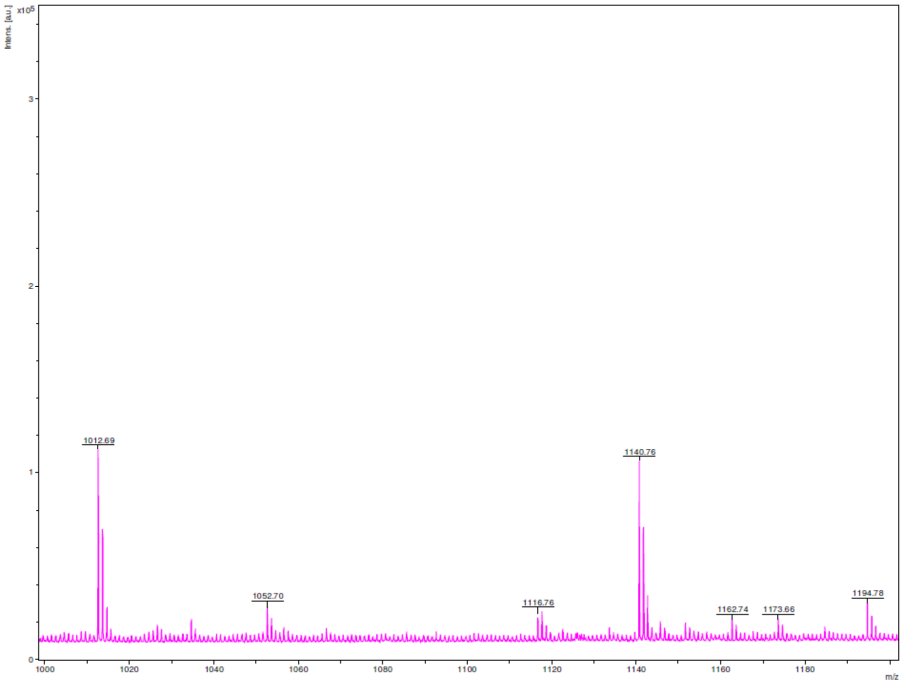	m/z<br>1012.69<br>1052.70<br>1116.76<br>1140.76<br>1162.74<br>1173.66<br>1194.78

<table>
<tr>
<td>X. miraniensis DSM 17902</td>
<td>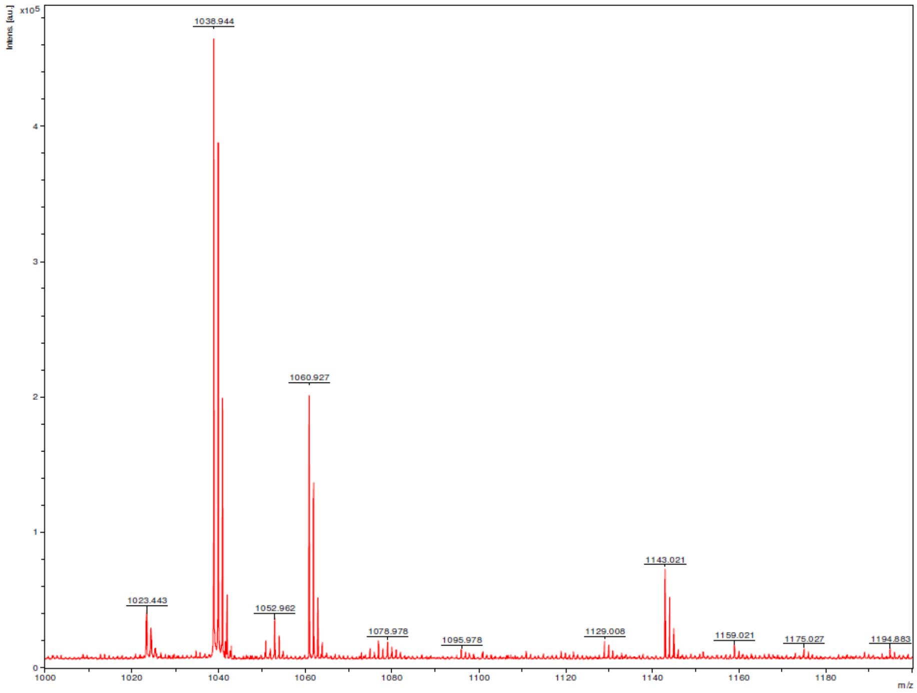

m/z
1023.443
1038.944
1050.945
1052.962
1060.927
1074.943
1076.898
1078.978
1095.978
1129.008
1143.021
1145.044
1159.021
1175.027
1194.883</td>
</tr>
<tr>
<td>X. poinarii G6</td>
<td>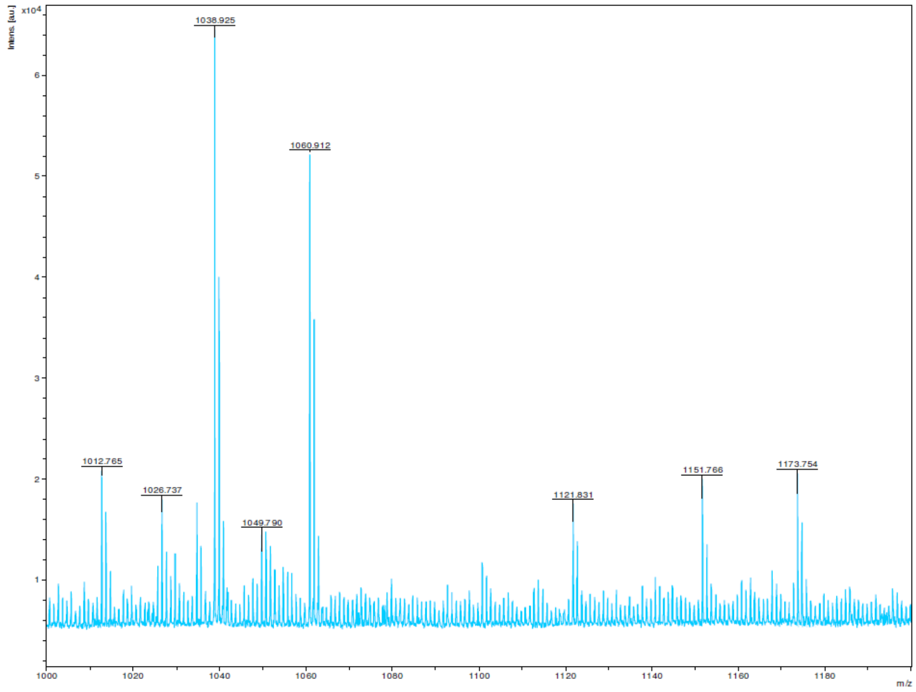

m/z
1012.765
1026.737
1029.752
1034.770
1038.925
1049.790
1054.739
1060.912
1121.831
1151.766
1173.754</td>
</tr>
</table>

X. vietnamensis DSM 22392	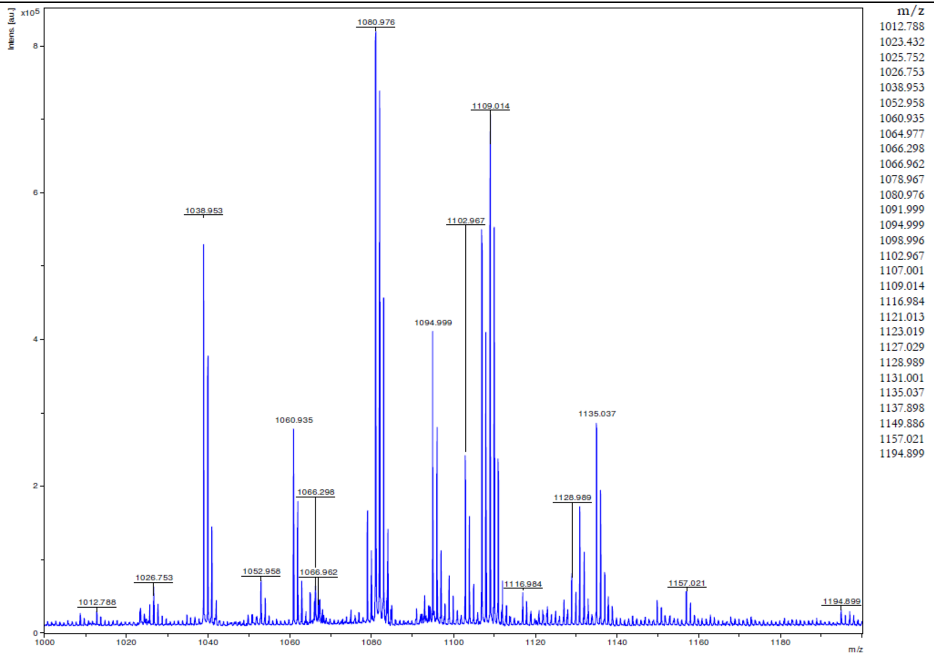

Table S4: Strains and plasmids used in this study.

Strain or plasmid	Genotype and relevant characteristics	Reference or source
**Strains**		
Xenorhabdus nematophila F1	Wild-type isolated from *Steinernema carpocapsae* nematode Plougastel (Brittany)	Laboratory collection
Xenorhabdus nematophila F1 variant 2	Secondary variant selected from *in vitro* cultures of *X. nematophila* F1 primary variant	(2)
Xenorhabdus nematophila Δ*paxA*	F1 *paxA* ::ΩCm	This study
Xenorhabdus nematophila Δ*ngrA*	F1 *ngrA* ::ΩCm	(3)
Xenorhabdus bovienii feltiae France	Isolated from *Steinernema feltiae*	Laboratory collection
Xenorhabdus budapestensis DSM 16342^{T}	Isolated from *Steinernema bicornutum*	(4)
Xenorhabdus cabanillasii DSM 17905^{T}	Isolated from *Steinernema riobravae*	(5)
Xenorhabdus hominickii DSM 17903^{T}	Isolated from *Steinernema karii*	(5)
Xenorhabdus ishibashii DSM 22670^{T}	Isolated from *Steinernema aciari*	(6)
Xenorhabdus mauleonii DSM 17908^{T}	Isolated from *Steinernema sp .*	(5)
Xenorhabdus miraniensis DSM 17902^{T}	Isolated from *Steinernema sp .* (lost)	(5)
Xenorhabdus poinarii G6^{T}	Isolated from *Steinernema glaseri*	(7)
Xenorhabdus vietnamensis DSM 22392^{T}	Isolated from *Steinernema sangi*	(8)
Escherichia coli XL1-Blue MRF'	Δ(*mcrA*)183 Δ(*mcrCB -hsdSMR -mrr*)173 *endA1 supE44 thi-1 recA1 gyrA96 relA1 lac [F' proAB lacIqZΔM15 Tn10(Tet^{r})]*	Stratagene
Escherichia coli WM3064	*thrB* 1004 *pro thi rpsl hsdS lac*ZΔM15 RP4-1360Δ(*araBAD*)567 Δ*dapA1341* ::[erm pir (wt)] donor strain	(9)
Micrococcus luteus CIP103430		(10)
Stenotrophomonas maltophilia ALL5	StmSc_ALL5, isolated from *S. carpocapsae* All (David Shapiro)	
Pseudomonas protegens PPSC10	Pp_Sc_PPSC10, isolated from *S. carpocapsae* SK27	
Ochrobactrum sp. ALL4	OchSc_ALL4, isolated from *S. carpocapsae* All (David Shapiro)	
Achromobacter sp. D7.1	AchSc_D7-1, Isolated from *S. carpocapsae* SK27	
Alcaligenes faecalis SC	AlcfSC_Sc, Isolated from *S. carpocapsae* SK27	(11)
Pseudochrobactrum sp. AL3	PochSc_AL3, isolated from *S. carpocapsae* All (David Shapiro)	
Brevundimonas sp. ALL3	Isolated from *S. carpocapsae* All (David Shapiro)	
Enteroccocus mundtii SP	Isolated from *S. littoralis espagnol* L6 stage	
Diutina rugosa GC riz	Isolated from *S. frugiperda* rice variant L6 stage	
**Plasmids**		
pJQ200SK	Gm^{r} *sacRB* mob oriV (p15A replicon) suicide plasmid	(3)
pHP45-ΩCam	pHP45 plasmid carrying the ΩCam interposon	(12)
pJQ-paxA:: ΩCm	pJQ suicide plasmid carrying the ΩCam interposon cloned between the two regions upstream and downstream of the *paxA* gene	This study

Table S5: Primers used in this study.

Used for	Primers	Sequences 5’-3’
paxA mutant construction	L-*paxA-SalI*	GCAGTCGACATGTTGCTGATCGCGTTATT
	R-*paxA-BamHI*	CGGGATCCTAACGCCCCGAAATAGAGTG
	L-*paxA-BamHI*	CGGGATCCGGCAAAGACTATCCGTCAGG
	R-*paxA-SpeI*	GCACTAGTAGTTGATTGGCACGTTGGTT
paxA mutant verification	L-*paxTA*	CGACCAATCTTTCAGCAGGT
	R-verif-*pax*	AACTCCGGCGTTGACAAG

References

1. Fuchs SW, Proschak A, Jaskolla TW, Karas M, Bode HB. 2011. Structure elucidation and biosynthesis of lysine-rich cyclic peptides in *Xenorhabdus nematophila*. *Org. Biomol. Chem.* 9(9):3130-3132. https://doi.org/10.1039/c1ob05097d.
2. Givaudan A, Baghdiguian S, Lanois A, Boemare N. 1995. Swarming and swimming changes concomitant with phase variation in *Xenorhabdus nematophilus*. *Appl Environ Microbiol* 61:1408–1413.
3. Lanois-Nouri A, Pantel L, Fu J, Houard J, Ogier JC, Polikanov YS, Racine E, Wang H, Gaudriault S, Givaudan A, Gualtieri M. 2022. The Odilorhabdin antibiotic biosynthetic cluster and acetyltransferase self-resistance locus are niche and species specific. *mBio* 13:e02826-21. https://doi.org/10.1128/mbio.02826-21.
4. Lengyel K, Lang E, Fodor A, Szállás E, Schumann P, Stackebrandt E. 2005. Description of four novel species of *Xenorhabdus*, family *Enterobacteriaceae*: *Xenorhabdus budapestensis* sp. nov., *Xenorhabdus ehlersii* sp. nov., *Xenorhabdus innexi* sp. nov., and *Xenorhabdus szentirmaii* sp. nov. *Syst. Appl. Microbiol.* 28(2):115-122. https://doi.org/10.1016/j.syapm.2004.12.003.
5. Tailliez P, Pagès S, Ginibre N, Boemare N. 2006. New insight into diversity in the genus *Xenorhabdus*, including the description of ten novel species. *Int. J. Syst. Evol. Microbiol.* 56(12):2805-2818. https://doi.org/10.1099/ijs.0.64287-0.
6. Kuwata R, Qiu LH, Wang W, Harada Y, Yoshida M, Kondo E, Yoshiga T. (2013). *Xenorhabdus ishibashii* sp. nov., isolated from the entomopathogenic nematode *Steinernema aciari*. *Int. J. Syst. Evol. Microbiol.* 63(Pt 5):1690-1695. https://doi.org/10.1099/ijs.0.041145-0.
7. Akhurst RJ. 1986. *Xenorhabdus nematophilus* subsp. *poinarii*: its interaction with insect pathogenic nematodes. *Syst. Appl. Microbiol.* 8(1):142-147.
8. Tailliez P, Laroui C, Ginibre N, Paule A, Pagès S, Boemare N. 2010. Phylogeny of *Photorhabdus* and *Xenorhabdus* based on universally conserved protein-coding sequences and implications for the taxonomy of these two genera. Proposal of new taxa: *X. vietnamensis* sp. nov., *P. luminescens* subsp. *caribbeanensis* subsp. nov., *P. luminescens* subsp. *hainanensis* subsp. nov., *P. temperata* subsp. *khanii* subsp. nov., *P. temperata* subsp. *tasmaniensis* subsp. nov., and the reclassification of *P. luminescens* subsp. *thracensis* as *P. temperata* subsp. *thracensis* comb. nov. *Int J Syst Evol Microbiol* 60:1921–1937. https://doi.org/10.1099/ijs.0.014308-0.
9. Paulick A, Koerdt A, Lassak J, Huntley S, Wilms I, Narberhaus F, Thormann KM. 2009. Two different stator systems drive a single polar flagellum in *Shewanella oneidensis* MR-1. *Mol. Microbiol.* 71(4):836-850. https://doi.org/10.1111/j.1365-2958.2008.06566.x.

10. Boemare N, Thaler JO, Lanois A. 1997. Simple bacteriological tests for phenotypic characterization of *Xenorhabdus* and *Photorhabdus* phase variants. *Symbiosis* 22:167–175.
11. Ogier JC, Pagès S, Frayssinet M, Gaudriault S. 2020. Entomopathogenic nematode-associated microbiota: from monoxenic paradigm to pathobiome. *Microbiome* 8:25. https://doi.org/10.1186/s40168-020-00800-5.
12. Fellay R, Frey J, Krisch H. 1987. Interposon mutagenesis of soil and water bacteria: a family of DNA fragments designed for in vitro insertional mutagenesis of Gram-negative bacteria. Gene 52: 147–154.
